# Zinc drives vasorelaxation by acting in sensory nerves, endothelium and smooth muscle

**DOI:** 10.1038/s41467-021-23198-6

**Published:** 2021-06-01

**Authors:** Ashenafi H. Betrie, James A. Brock, Osama F. Harraz, Ashley I. Bush, Guo-Wei He, Mark T. Nelson, James A. Angus, Christine E. Wright, Scott Ayton

**Affiliations:** 1 Melbourne Dementia Research Centre, Florey Institute of Neuroscience and Mental Health, The University of Melbourne, Victoria, Australia; 2 Cardiovascular Therapeutics Unit, Department of Biochemistry and Pharmacology, The University of Melbourne, Victoria, Australia; 3 Department of Cardiovascular Surgery & Center for Basic Medical Research, TEDA International Cardiovascular Hospital, Chinese Academy of Medical Sciences; The Institute of Cardiovascular Diseases, Tianjin University, Tianjin; Center for Drug Development, Wannan Medical College, Wuhu, Anhui China; 4 Department of Anatomy and Physiology, The University of Melbourne, Victoria, Australia; 5 Department of Pharmacology, Larner College of Medicine, University of Vermont, Burlington, Vermont USA; 6 Vermont Center for Cardiovascular and Brain Health, Larner College of Medicine, University of Vermont, Burlington, VT USA; 7 Institute of Cardiovascular Sciences, University of Manchester, Manchester, UK

**Keywords:** Pharmacology, Circulation, Cardiovascular biology

## Abstract

Zinc, an abundant transition metal, serves as a signalling molecule in several biological systems. Zinc transporters are genetically associated with cardiovascular diseases but the function of zinc in vascular tone regulation is unknown. We found that elevating cytoplasmic zinc using ionophores relaxed rat and human isolated blood vessels and caused hyperpolarization of smooth muscle membrane. Furthermore, zinc ionophores lowered blood pressure in anaesthetized rats and increased blood flow without affecting heart rate. Conversely, intracellular zinc chelation induced contraction of selected vessels from rats and humans and depolarized vascular smooth muscle membrane potential. We demonstrate three mechanisms for zinc-induced vasorelaxation: (1) activation of transient receptor potential ankyrin 1 to increase calcitonin gene-related peptide signalling from perivascular sensory nerves; (2) enhancement of cyclooxygenase-sensitive vasodilatory prostanoid signalling in the endothelium; and (3) inhibition of voltage-gated calcium channels in the smooth muscle. These data introduce zinc as a new target for vascular therapeutics.

## Abstract (web summary)

Metals such as calcium and potassium have long been known to regulate the diameter of arteries that control blood flow. Here, we report that zinc causes relaxation of blood vessels and reduces blood pressure by its coordinated action in sensory nerves, endothelium and smooth muscle cells.

## Introduction

The understanding of the roles of the S-block metals, calcium, and potassium, in the maintenance of cardiovascular physiology has led to the discovery of important drugs for the treatment of cardio- and cerebrovascular diseases. Zinc is an abundant transition metal that is essential for many proteins that serve structural, functional, and signaling functions in cardiovascular biology. For example, nitric oxide synthase, phosphodiesterase, angiotensin-converting enzyme, superoxide dismutase, neprilysin, and angiotensin II either directly bind zinc or their activities are dependent on zinc^1–4^. Further, zinc modulates the activity of ion channels in the calcium, potassium, and transient receptor potential channel families in different cell types^5–10^. For example, zinc potently activates transient receptor potential cation channel subfamily A (ankyrin) member 1 (TRPA1) channel^7,8^ by the influx of zinc through the channel that then binds to intracellular cysteine and histidine residues to activate it^8^. The activation of TRPA1 channels, which are expressed in perivascular sensory nerves, stimulates the release of CGRP to cause vasodilation^11–16^.

Despite the ability of zinc to regulate the activity of TRPA1 as well as other ion channels and proteins important in vascular physiology, the significance of zinc in vascular tone regulation is not well understood. A recent meta-analysis of randomized controlled trials of zinc supplementation showed that zinc decreased systolic blood pressure^17^. Zinc deficiency on the other hand has been linked with high blood pressure in animal models^18,19^. However, reported associations between serum zinc levels and blood pressure are inconsistent^20,21^, which may reflect the poor correlation between serum and tissue levels of zinc^22,23^.

Cytoplasmic zinc is increasingly appreciated for serving signaling functions^5,24,25^. Accordingly, cytoplasmic zinc is tightly regulated by 14 zinc-/iron-regulated transporters (ZIPs) that import zinc into the cytoplasm and 10 zinc transporters (ZnTs) that efflux zinc out of the cytoplasm^23^. ZIP12 was recently reported to regulate pulmonary vascular responses to hypoxia^26^, while a mutation in ZnT9 was reported to cause cerebro-renal syndrome with hypertension^27^. ZIP8 and ZIP13 were identified in genome-wide association studies of blood pressure^28,29^, but how zinc may affect blood pressure or vascular tone remains unknown.

Here we demonstrate a role for zinc in vascular tone regulation. Since there are currently no compounds known to modulate the activity of zinc transporters, and the expression profiles and importance of these zinc transporters in different cell types and vascular beds have not been determined, we used ionophores and cell-permeable chelators, which bypass the complex zinc transport mechanisms, as probes to examine the role of zinc in vascular physiology. We report that elevating cytoplasmic zinc using ionophores causes relaxation to animal and human isolated vessels and these compounds lower blood pressure in rats and mice. Conversely, lowering cytoplasmic zinc with chelators causes vasoconstriction. We demonstrate using pharmacological, electrophysiological, and genetic approaches that zinc signals vasorelaxation by its action in the perivascular sensory nerve, endothelium, and the vascular smooth muscle. We, therefore, introduce zinc as an important metal ion that complements the actions of calcium^30^ and potassium^31^ in the regulation of vascular tone that have been appreciated for more than half a century.

## Results

### Zinc ionophores cause vasorelaxation and decrease blood pressure

**Fig. 1:**
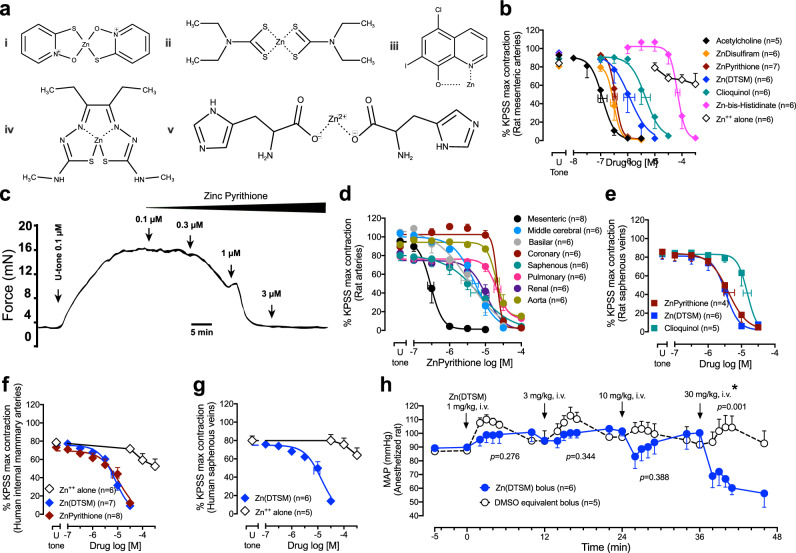
Zinc ionophores cause vasorelaxation of rat and human isolated arteries and decrease blood pressure. **a** Chemical structures of the zinc ionophores used: zinc pyrithione (**i**), zinc disulfiram (**ii**), clioquinol (**iii**), Zn(DTSM) (**iv**), and zinc-bis-histidinate (**v**). **b** Relaxation responses to zinc ionophores in rat mesenteric arteries contracted with the thromboxane-mimetic, U46619 (U tone). A representative trace showing the effects of zinc pyrithione on a U-contracted rat mesenteric artery (**c**) and averaged data in different arteries (**d**). **e** Relaxation responses to zinc ionophores in rat saphenous vein. **f**, **g** Relaxation of human isolated internal mammary arteries (**f**) and saphenous veins (**g**) by selected zinc ionophores. **h** In vivo depressor effects of successive, escalating-dose bolus intravenous injections of Zn(DTSM) on mean arterial pressure (MAP) in anaesthetized rats. In **b** and **d**–**g** the contractions are presented as a % of the KPSS (124 mM K^+^)-evoked contractions (Supplementary Table 1). Error bars are SEM (those not shown are contained within the symbol); horizontal error bars are for the average EC_50_. *n*, number of rats or arteries isolated from separate rats/humans. *Statistically significant, two-tailed unpaired Student’s *t* test (T(8) = 5.032) at 5 min compared to the equivalent dose of vehicle.

Administration of five structurally dissimilar zinc-delivery agents, zinc pyrithione, zinc disulfiram, clioquinol, Zn(DTSM), and zinc-bis-histidinate (Fig. 1a), caused concentration-dependent relaxation of rat mesenteric arteries contracted with the thromboxane mimetic, U46619 (Fig. 1b, Supplementary Table 1). Similar results were found when arteries were contracted with high potassium (62 mM, Supplementary Fig. 1a), endothelin-1 (Supplementary Fig. 1b) or electrical nerve stimulation (Supplementary Fig. 1c) with varying potencies. The exemplar ionophores, zinc pyrithione and Zn(DTSM), were broad-acting as they relaxed vessels isolated from anatomically disparate regions of rats (mesenteric, cerebral, coronary, saphenous, pulmonary, renal, and aorta, Fig. 1c, d and Supplementary Fig. 2). The potency and efficacy of zinc pyrithione and zinc disulfiram were comparable to the classical relaxant agent acetylcholine (Fig. 1b, Supplementary Table 1) in rat mesenteric arteries. These effects were also translatable to humans, with both zinc pyrithione and Zn(DTSM) causing a concentration-dependent relaxation of human internal mammary arteries and saphenous veins (Fig. 1f, g). In the absence of an ionophore, extracellular zinc had minimal effects, indicating that the vasorelaxation was attributable to intracellular zinc (Fig. 1b, f, g; Supplementary Fig. 1a, c). These effects were also relevant in vivo. In anesthetized rats, intravenous Zn(DTSM) dose-dependently lowered mean arterial blood pressure (Fig. 1h) and decreased hindquarter vascular resistance, without changing heart rate (Supplementary Fig. 3a, b).

### Chelation of intracellular zinc causes contraction of selected vessels

**Fig. 2:**
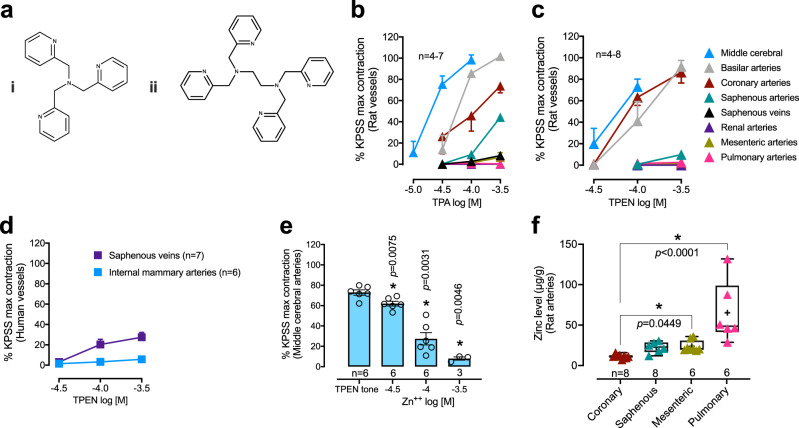
Zinc chelators contract selected rat and human isolated arteries. **a** Chemical structures of the cell-permeable zinc chelators used: TPA (**i**) and TPEN (**ii**). **b**–**d** Selective contractile effects of the zinc-selective chelators, TPA (**b**) and TPEN (**c**) on resting (basal) tone in vessels from rats and humans (**d**). **e** Zinc added after contraction of middle cerebral arteries with TPEN (100 µM) caused reversal of the effect. **f** Zinc levels per wet weight in rat arteries. Whiskers are min to max values and boxes are 25–75th percentile, where the line inside the box is the median and the + sign is the mean. Responses in **b**–**e** are %KPSS. Error bars are SEM (those not shown are contained within the symbol). *n*, number of arteries isolated from individual rats/humans. *Statistically significant, mixed-effects 1-way ANOVA [*F*(1.55, 6.2) = 62.81, *p* = 0.0001] with Dunnett’s post-test compared to TPEN tone in **e** and Kruskal–Wallis test [*H*(3) = 20.97, *p* = 0.0001] with Dunn’s post-test in (**f**).

To determine whether endogenous zinc affects vascular tone, we challenged isolated arteries with cell-permeable, zinc-selective chelators: tris(2-pyridylmethyl)amine (TPA) or N,N,N′,N′-tetrakis(B-pyridylmethyl)ethylenediamine (TPEN, Fig. 2a). Both chelators induced contraction of rat isolated middle cerebral, basilar, coronary and saphenous arteries (Fig. 2b, c). The contraction by TPEN (100 µM) in middle cerebral arteries was abolished by zinc coadministration (Fig. 2e) demonstrating zinc-dependency. In contrast, neither TPA nor TPEN caused contraction of mesenteric, renal or pulmonary arteries. TPEN also caused significant contraction of human saphenous veins (20–30% KPSS), but not human internal mammary arteries (Fig. 2d). Given these regional differences to zinc chelators, we hypothesized that arteries that were more susceptible to chelation-mediated contraction may be those with lower zinc content. Indeed, the zinc chelator-responsive rat coronary arteries contained lower zinc (but similar copper and iron levels) than mesenteric and pulmonary arteries (Fig. 2f, Supplementary Fig. 4; cerebral arteries were not large enough to allow accurate measurement of metals).

### Cytoplasmic zinc enhances CGRP signaling from perivascular sensory nerves

**Fig. 3:**
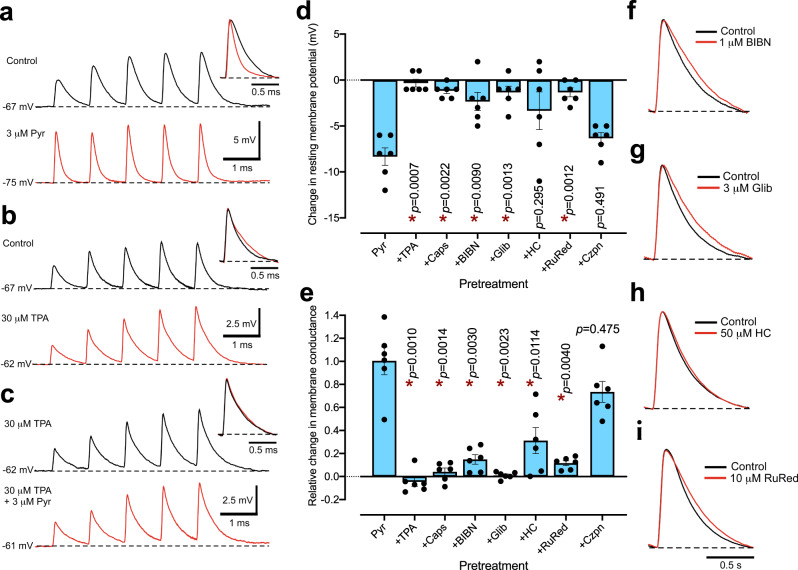
Zinc modulates vascular smooth muscle conductance and membrane potential via CGRP signaling from rat mesenteric artery sensory nerves. **a**–**c** Representative traces of EJPs in individual tissues stimulated with trains of five stimuli at 1 Hz. The zinc ionophore, pyrithione (Pyr, 3 µM) produced hyperpolarization and accelerated the decay time of EJPs (**a**), whereas the zinc chelator TPA (30 µM) induced the reverse response (**b**) and neutralized the effects of pyrithione (**c**). Insets are amplitude-normalized, expanded overlaid traces of the 5th EJP with/without treatment. **d**, **e** Changes in resting membrane potential (**d**) and membrane conductance (calculated from the 5th EJP; **e**) caused by pyrithione alone and with the following pretreatment: TPA (30 µM, zinc chelation), capsaicin (Caps; 10 µM; sensory nerve desensitization), BIBN4096 (BIBN; 1 µM; inhibition of CGRP receptors), glibenclamide (Glib; 3 µM; blockade of K_ATP_ channels), HC030031 (HC; 50 µM; inhibition of TRPA1) or ruthenium red (RuRed; 10 µM; inhibition of TRPA1), and capsazepine (Czpn; 10 µM; inhibition of TRPV1). Error bars are SEM. *Statistically significant, Brown–Forsythe–Welch ANOVA [*F*(7, 13.93) = 8.659, *p* = 0.0004 in (**d**) and *F*(7, 18.36) = 28.63, *p* < 0.0001 in (**e**)] with Dunnett T3 post-test vs. pyrithione. **f**–**i** Effects of pretreatment alone on EJP time course. **d**, **e** *n* = 6 arteries from separate rats.

To test the mechanisms by which zinc causes vasorelaxation, we systematically investigated the contribution of changes in smooth muscle membrane potential and ion conductance, and of perivascular sensory nerves and endothelium that can release vasodilatory mediators in response to an increase in cytoplasmic zinc. We used intracellular recording, and measurement of purinergic excitatory junction potentials (EJPs) to assess the role of cytoplasmic zinc in smooth muscle membrane potential. Rat mesenteric artery smooth muscle cells (SMCs) were hyperpolarized by the exemplar zinc ionophores, pyrithione and Zn(DTSM), (3 µM, Fig. 3a, d, Supplementary Fig. 5a), but not extracellular zinc alone (100 µM, Supplementary Table 2), and were depolarized by the intracellular chelators TPA and TPEN (30 µM, Fig. 3b, Supplementary Table 2). The time constant of decay of EJPs was decreased by zinc ionophores and increased by zinc chelators, indicative of a rise and fall in membrane conductance, respectively (Fig. 3a, b, e; Supplementary Table 2). The ionophore-induced hyperpolarization was neutralized by intracellular zinc chelation (Fig. 3c, d, Supplementary Fig. 5b). Thus, cytoplasmic zinc acts through modulation of K^+^ permeability to play a physiological role in regulating membrane potential.

We noted that zinc ionophore-mediated effects on membrane potential, EJPs, and vascular tone were similar to those produced by calcitonin gene-related peptide (CGRP) and activation of the perivascular sensory nerves with capsaicin^32^. Likewise, the effects of intracellular zinc chelators were similar to those of CGRP receptor inhibition by BIBN4096 and blockade of ATP-sensitive potassium channels (K_ATP_) (Fig. 3f, g), activation of which produces CGRP-induced hyperpolarization^32,33^. We found that capsaicin pretreatment (which desensitized sensory nerves), blockade of CGRP receptors (BIBN4096, 1 µM), or K_ATP_ channel inhibition (glibenclamide, 3 µM) all prevented the smooth muscle hyperpolarization as well as the increase in membrane conductance induced by zinc ionophores (Fig. 3d, e; Supplementary Fig. 5c, d). These findings confirm that cytoplasmic zinc stimulates CGRP signaling. The TRPA1 inhibitors HC030031 (50 µM) and ruthenium red (10 µM), but not the selective TRPV1 antagonist capsazepine (10 µM), attenuated the effects of zinc pyrithione on membrane potential suggesting the involvement of TRPA1 but not TRPV1 channels.

**Fig. 4:**
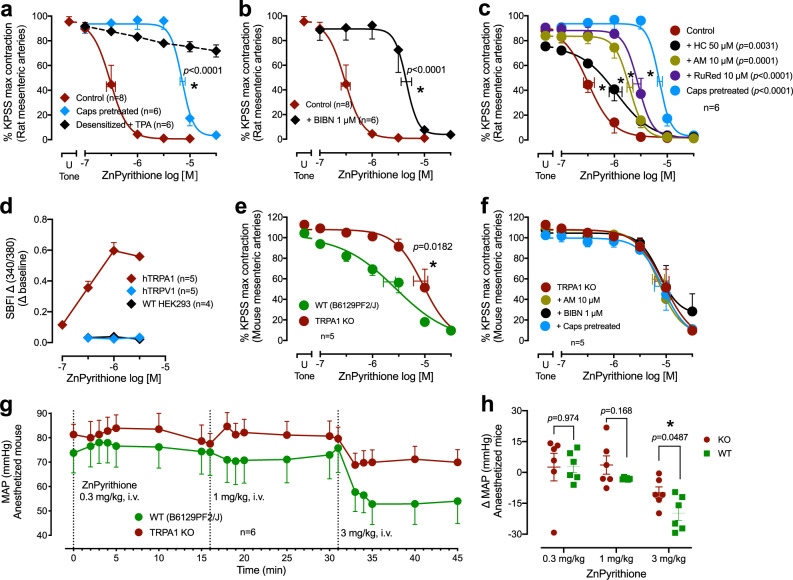
Impact of perivascular sensory nerves on zinc ionophore-dependent vasorelaxation in rats and TRPA1 KO mice. **a**–**c** Effects of zinc pyrithione with/without perivascular sensory nerve desensitization by capsaicin (caps pretreated), or concomitant zinc chelation with TPA (**a**), the CGRP receptor antagonist BIBN4096 (BIBN, **b**), or the TRPA1 channel blockers ruthenium red (RuRed), AM0902 (AM) or HC030031(HC) (**c**). **d** Zinc pyrithione activates hTRPA1 expressed in HEK293 cells (measured by the Na^+^ selective intracellular dye SBFI-AM), but not hTRPV1 or non-transfected (WT) HEK293 cells. **e** Decreased relaxation potency of zinc pyrithione in isolated mesenteric arteries from TRPA1 KO mice compared to WT mice. **f** Capsaicin desensitization, BIBN4096 or AM0902 did not affect the relaxation potency of zinc pyrithione in TRPA1 KO mice. **g**, **h** Mean arterial pressure (MAP) after an intravenous (i.v.) bolus injections of 0.3, 1, and 3 mg/kg zinc pyrithione (**g**) and the peak change in MAP (**h**) in WT (B6129PF2/J) mice. Arteries in **a**–**c**, **e**, and **f** were contracted with U46619 (U Tone) and responses are expressed as a % of the KPSS (124 mM K^+^)-evoked contractions (Supplementary Table 3). Error bars are SEM (those not shown are contained within the symbol); horizontal error bars are for the average EC_50_. *n*, number of rats, mice or arteries isolated from separate animals. The control curve in (**a**) and the TRPA1 KO curve in (**e**) are shown again in (**b**) and (**f**), respectively for representation. *Statistically significant, 1-way ANOVA of EC_50_ [*F*(9,61) = 27.60, *p* < 0.0001 in (**a**, **b**) and *F*(3,20) = 15.15, *p* < 0.0001 in (**c**)] followed by Dunnett’s post-test vs. control; *F*(3,19) = 0.0538, *p* = 0.983 in (**d**); two-tailed unpaired Student’s *t* test [T(9) = 2.879 in (**e**) and T(10) = 2.444 in (**h**)].

Sensory nerve involvement in the vasorelaxant mechanism of zinc ionophores was confirmed in isolated arteries. Desensitization of sensory nerves (with capsaicin 10 µM pretreatment followed by washout, Fig. 4a; Supplementary Fig. 6a–c) or CGRP receptor inhibition (with BIBN4096 1 µM, a concentration that inhibited the vasorelaxation to CGRP Fig. 4b, Supplementary Fig. 7) markedly decreased the vasorelaxant potency of the ionophores. To identify if this mechanism was physiologically relevant in vivo, we investigated the impact of intravenous (i.v.) injection of a physiological ionophore, water-soluble zinc-bis-histidinate (Fig. 1a), on cutaneous blood flow in the hind paw of anesthetized rats. Previous studies have demonstrated that selective activation of the sensory nerves causes vasodilation in the hindpaw^34^. Zinc-bis-histidinate (3, 10 and 30 mg/kg i.v.), but not histidine alone (67 mg/kg i.v., Supplementary Fig. 8a, b), caused an increase in cutaneous blood flow measured by laser Doppler but without changing arterial blood pressure (Supplementary Fig. 8c). The increase in cutaneous flow was dependent on sensory nerve activity since the inhibition of CGRP receptors with BIBN4096 pretreatment (at a dose that blocks sensory nerve stimulation-induced vasodilatation^35^, 3 mg/kg i.v.) attenuated the effect (Supplementary Fig. 8b).

### Zinc activates TRPA1 channels to stimulate CGRP signaling from sensory nerves

Cytoplasmic zinc activates TRPA1 in somatosensory neurons^7,8^. The activation of the two main TRP channels expressed in perivascular sensory nerves, TRPA1 and TRPV1, has been shown to evoke the influx of calcium and stimulate the release of CGRP to cause vasodilation^11–16^. Thus, we hypothesized that the activation of TRPA1 channels by cytoplasmic zinc would stimulate the release of dilatory neuropeptides from perivascular sensory nerves to cause vasodilation. Accordingly, we found that TRPA1 inhibition with non-selective ruthenium red or selective AM0902 and HC030031, but not TRPV1-selective inhibitor capsazepine, attenuated the zinc ionophore-induced vasorelaxation (Fig. 4c). Ruthenium red and HC030031 also inhibited the zinc ionophores-induced hyperpolarization (Fig. 3d, e, h, i) and vasorelaxation (Fig. 4e).

To further confirm that cytoplasmic zinc selectively activates TRPA1 but not TRPV1 channels, we employed HEK293 cells expressing tetracycline-inducible human TRPA1 or TRPV1 channels and monitored the activity of the channels by measuring intracellular cation levels. TRPA1 and V1 channels are permeable to both calcium and sodium^7,36,37^. Although the most common cation monitored in these assays is calcium, calcium fluorophores are sensitive to zinc^38^, precluding their use for our purpose. Instead, we measured sodium influx to monitor the activity of TRPA1 and TRPV1 channels using the SBFI-AM fluorescent reporter and assessed the effect of increasing cytoplasmic zinc. Zinc pyrithione evoked concentration-dependent sodium influx in HEK293 cells expressing hTRPA1, but not hTRPV1 (Fig. 4d, positive controls: Supplementary Fig. 9).

Further evidence for the role of the TRPA1 channel was found in TRPA1 homozygous knockout (KO) mice where zinc pyrithione showed a decreased relaxation potency in isolated mesenteric arteries compared to WT (B6129PF2/J) mice (Fig. 4e). Capsaicin desensitization of perivascular sensory nerves, inhibition of CGRP receptor by BIBN, or inhibition of TRPA1 channels with the selective inhibitor AM0902, did not show any further change in the zinc ionophore relaxation potency in mesenteric arteries of TRPA1 KO mice (Fig. 4f). This suggests that other TRP channels do not contribute to the perivascular sensory nerve-mediated effect. In vivo, TRPA1 KO mice similarly showed less decrease in mean arterial blood pressure after bolus injection of increasing doses of zinc pyrithione compared to WT controls (peak change, −10 ± 3 mmHg in KO Vs −20 ± 3 mmHg in WT at 3 mg/kg; Fig. 4g, h), without a change in heart rate (Supplementary Fig. 10). Therefore, cytoplasmic zinc activates TRPA1 channels in perivascular sensory nerves eliciting the release of CGRP that causes vasorelaxation.

### Zinc-dependent vasorelaxant mechanisms beyond sensory nerves

**Fig. 5:**
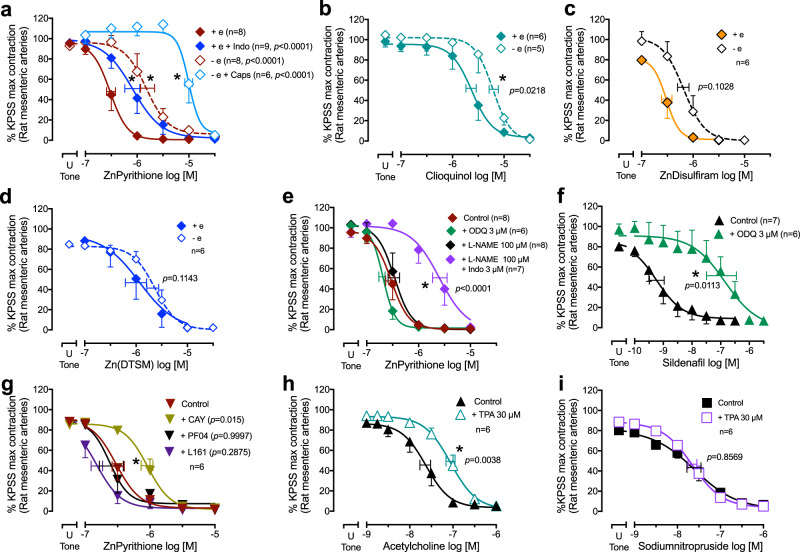
Effects of endothelium-dependent mechanisms on zinc ionophore-dependent vasorelaxation. **a** Effects of zinc pyrithione with/without endothelium (+/− e), cyclooxygenase inhibition (indomethacin, Indo 3 µM), and capsaicin desensitization (caps, 10 µM followed by washout). **b**–**d** Impact of endothelial denudation on zinc-dependent relaxation by clioquinol (**b**), zinc disulfiram (**c**), or Zn(DTSM) (**d**). **e** Inhibition of nitric oxide synthase with L-NAME (100 µM, *p* > 0.9999) or soluble guanylate cyclase with ODQ (3 µM, *p* = 0.9605) had no effect, but the combination of L-NAME and indomethacin decreased the potency of zinc pyrithione in endothelium-intact arteries. **f** Sildenafil-mediated relaxation potency was decreased by ODQ. **g** Inhibition of the prostacyclin IP receptor by the selective antagonist CAY10441 (CAY, 1 µM) but not the antagonists of EP_2_ receptor (PF-04418948, 1 µM) or the prostanoid EP_4_ receptor (L-161982, 1 µM) decreased the potency of zinc pyrithione. **h**, **i** Removal of endogenously available zinc by the selective chelator TPA (30 µM) caused a decrease in the relaxation potency of the nitric oxide-dependent acetylcholine (**h**), but not the nitric-oxide donor sodium nitroprusside (**i**). Arteries were contracted with U46619 (U Tone) and responses are expressed as % of the KPSS (124 mM K^+^)-evoked contractions (Supplementary Table 1). Error bars are SEM (those not shown are contained within the symbol); horizontal error bars are the average EC_50_. *n*, number of rats or arteries isolated from separate rats. The control curve in (**a**) is shown again in (**e**) for representation. *Statistically significant, two-tailed unpaired Student’s *t* test of EC_50_ (T(9) = 2.769 in (**b**), T(11) = 3.039 in (**f**), T(10) = 3.754 in (**h**)); 1-way ANOVA of EC_50_ [*F*(9,61) = 27.60, *p* < 0.0001 in (**a**, **e**) and *F*(3,20) = 3.892, *p* = 0.0243 in (**g**)] followed by with Dunnett’s post-test.

While inhibitors of CGRP signaling abolished the zinc ionophore-induced electrophysiological effects (Fig. 3d, e), they only attenuated the vasorelaxant potency of these agents without significant impact on the maximum efficacy (Fig. 4a–f). Thus, cytoplasmic zinc elevation may evoke vasorelaxation by additional mechanisms outside of sensory nerves. Removal of the endothelium decreased the relaxation potency of zinc pyrithione and clioquinol (Fig. 5a, b), but only showed a trend of inhibition of the potency of zinc disulfiram and did not impact Zn(DTSM) (Fig. 5c, d). To further explore the endothelium-dependent vasorelaxant mechanisms involved, rat isolated endothelium-intact mesenteric arteries were pretreated with inhibitors of nitric oxide signaling (L-NAME, 100 µM, to inhibit nitric oxide synthase or ODQ, 3 µM, to inhibit soluble guanylate cyclase), an inhibitor of prostanoid synthesis by cyclooxygenase (indomethacin, 3 µM) or inhibitors of endothelium-dependent hyperpolarization (apamin 50 nM + charybdotoxin 50 nM) before performing relaxation curves to zinc pyrithione. Only the inhibition of prostanoid synthesis with the cyclooxygenase inhibitor, indomethacin (Fig. 5a) decreased zinc pyrithione potency whereas inhibition of nitric oxide signaling (with L-NAME or ODQ, Fig. 5e, f), or of endothelium-dependent hyperpolarization (with apamin/charybdotoxin) did not (Supplementary Table 3). The combination of L-NAME and indomethacin pretreatment caused a modest (two to threefold) and nonsignificant inhibition of zinc pyrithione-induced relaxation (*p*EC_50_: 5.67 ± 0.14 when combined vs. 6.09 ± 0.14 with indomethacin alone, Fig. 5e). These results reveal a role for zinc in the production of dilatory prostanoids, although this action was not observed for all ionophores.

We further investigated the specific prostanoid responsible for the zinc-mediated vasorelaxation by using selective receptor antagonists for the prostacyclin IP receptor (CAY10441 also known as RO-1138452, 1 µM), the prostanoid EP_2_ receptor (PF-04418948, 1 µM) or the prostanoid EP_4_ receptor (L-161982, 1 µM). Only pretreatment with CAY10441 showed a decrease in the relaxation potency of zinc pyrithione (Fig. 5g) demonstrating the role of prostacyclin.

These results suggest that nitric oxide does not contribute significantly to the vasodilatory mechanism evoked by elevating cytoplasmic zinc with zinc pyrithione. However, zinc is a known cofactor of the enzyme nitric oxide synthase^2^, therefore we assessed whether the removal of freely accessible “labile” zinc would affect nitric oxide-mediated relaxation. Accordingly, pretreatment with the zinc-selective TPA (30 µM) caused a significant decrease in the relaxation potency of acetylcholine (which causes vasorelaxation by activating nitric oxide synthase; Fig. 5h) but not sodium nitroprusside (which directly delivers nitric oxide; Fig. 5i). These results confirmed that although zinc is important for the activity of nitric oxide synthase, the addition of excess cytoplasmic zinc by using ionophores does not further increase the activity of this enzyme.

When both sensory nerve desensitization and endothelial removal were performed, zinc pyrithione at high concentrations still caused maximal vasorelaxation (Fig. 5a). This vasorelaxant effect of zinc pyrithione is attributable to its ionophore activity since it was neutralized by zinc chelation (Fig. 4a). Therefore, we hypothesized an additional vasorelaxant signal for cytoplasmic zinc, possibly intrinsic to the smooth muscle.

### Vasorelaxant mechanisms of zinc in smooth muscle

Potassium channels are the primary regulators of the resting membrane potential in SMCs. While zinc has been reported to activate some potassium channels^6,9,39^, we found that inhibitors of K_ATP_ (glibenclamide (3 µM)), of calcium-activated potassium channels (BK_Ca_, combination of apamin (50 nM) and charybdotoxin (50 nM)) or of voltage-gated potassium channels (K_V,_ 4-aminopyridine (3 mM)) did not affect the potency or efficacy of zinc ionophore-induced vasorelaxation in rat isolated mesenteric arteries (Supplementary Fig. 11). The observation that glibenclamide blocks the hyperpolarizing effects of zinc pyrithione (Fig. 3d, e; Supplementary Fig. 5c, d), but not the vasorelaxation is similar to the findings for CGRP (Supplementary Fig. 7)^32,40^.

**Fig. 6:**
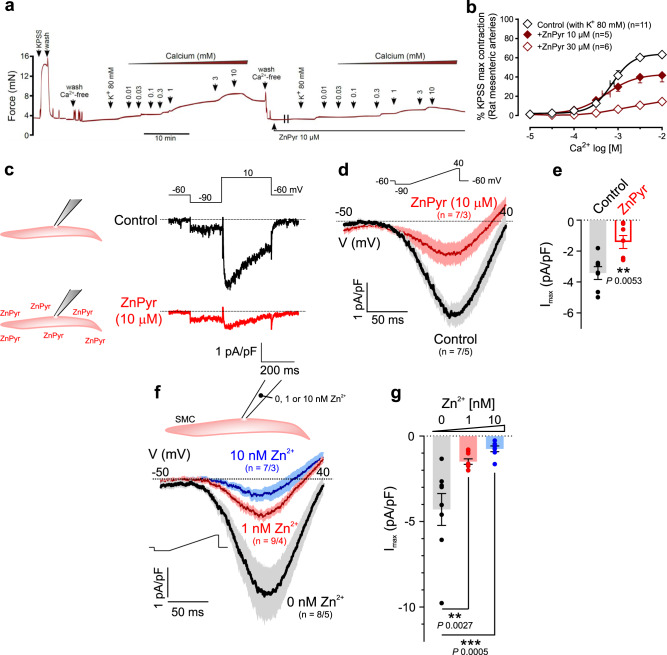
Effects of zinc on voltage-gated calcium channel (VGCC) current. **a**, **b** Effects of zinc pyrithione (ZnPyr) on depolarization-dependent (80 mM KCl), calcium-induced contraction in rat isolated mesenteric arteries. **a** Representative trace. **b** Averaged data (%KPSS). *n*: arteries from separate rats. **c**–**e** VGCC current (charge carrier: Ba^2+^) recorded in mouse freshly isolated mesenteric artery SMCs using the conventional whole-cell configuration with/without zinc pyrithione (10 μM; ZnPyr). **c** Representative traces obtained using a test step (10 mV, inset) with/without ZnPyr. **d**, **e** Averaged traces of inward currents evoked by a voltage ramp protocol (upper inset, **d**) and the maximum current density obtained in these experiments (*I*_max_, **e**) with/without 10 μM ZnPyr; **p < 0.01 two-tailed unpaired *t* test. **f**, **g** Effects of zinc delivered via the patch pipette on VGCC currents in mouse mesenteric artery SMCs. **f** Averaged current traces and **g** maximum current density (*I*_max_) of VGCC currents recorded using ramp protocol with/without 1 or 10 nM free Zn^2+^ in the pipette solution. **p < 0.01, ***p < 0.001, 1-way ANOVA [*F*(2,21) = 11.08, *p* = 0.0005) with Dunnett’s post-test. Error bars are SEM; horizontal error bars are for the average EC_50_. *n*/*n*: number of cells/number of mice.

To test whether zinc ionophores impact calcium signaling involved in smooth muscle contraction, we determined calcium concentration-response relationships in rat isolated mesenteric arteries incubated in a calcium-free buffer and depolarized with high potassium (80 mM) to activate voltage-gated calcium channel (VGCC) currents. Zinc ionophores (Fig. 6a, b; Supplementary Fig. 12a–c), but not extracellular zinc (Supplementary Fig. 12d), inhibited calcium-induced contraction, in a concentration-dependent manner consistent with previous reports that intracellular zinc inhibits high- and low-voltage-activated calcium channels in other cell types^5,41^. To definitively assess the contribution of VGCC, we employed whole-cell patch–clamp electrophysiological recordings in mouse freshly isolated mesenteric artery SMCs, using barium (10 mM) as the charge carrier. Zinc pyrithione inhibited VGCC currents (Fig. 6c–e). To confirm cytoplasmic zinc was responsible for this effect of zinc pyrithione, we directly introduced zinc into the cytoplasm using the patch pipette. Zinc caused a potent, concentration-dependent inhibition of VGCC current (53% inhibition with 1 nM and 72% with 10 nM) without changing the time constants of activation or inactivation confirming that the increase in cytoplasmic zinc produced by zinc ionophores inhibited VGCCs (Fig. 6f, g; Supplementary Fig. 13).

## Discussion

**Fig. 7:**
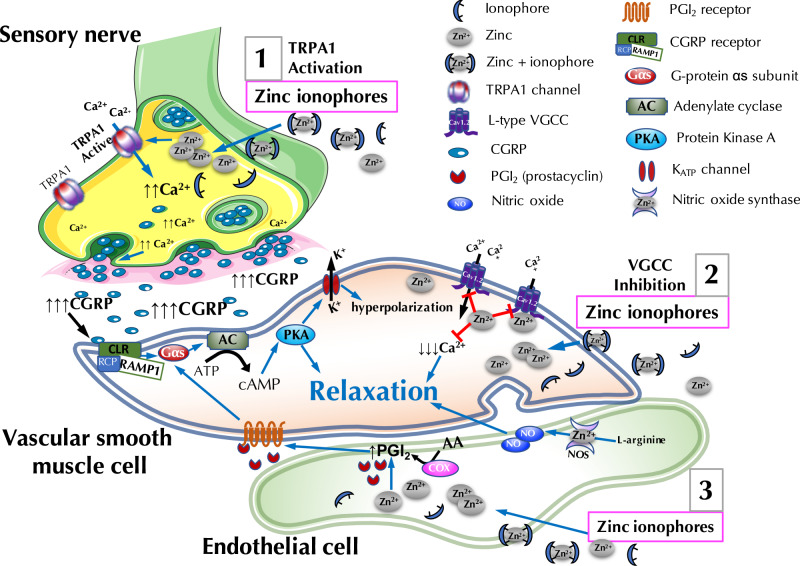
Mechanism of zinc-mediated vasorelaxation. **1** Zinc in the cytoplasm of perivascular sensory nerves activates transient receptor potential ankyrin 1 (TRPA1) that causes calcium influx and, in turn, releases calcitonin gene-related peptide (CGRP). CGRP binds to its G-protein coupled receptor (CLR-RAMP1-RCP) on the smooth muscle to cause cAMP-dependent hyperpolarization and relaxation. **2** Vascular smooth muscle cells are relaxed by cytoplasmic zinc that inhibits voltage-gated calcium channels (VGCC). **3** In the endothelium, increased cytoplasmic zinc using pyrithione and clioquinol causes relaxation of the smooth muscle by increasing dilatory prostanoid signaling. Nitric oxide synthase (NOS) function requires zinc, but its activity is not stimulated by excess zinc, rather it is inhibited by zinc deprivation. Image partly created with BioRender.com.

In this study, we demonstrate a role for zinc in vascular tone regulation. Although such a role is predicted by genetic findings linking zinc transporters to cardiovascular diseases including hypertension^26–29^, the role of zinc in the mechanisms regulating blood vessel tone has not been described. Here, we show that cytoplasmic zinc causes vasorelaxation via three mechanisms: (1) TRPA1-mediated activation of CGRP release from perivascular sensory nerves; (2) increased dilatory prostanoid signaling from the endothelium; and (3) inhibition of VGCC in vascular smooth muscle (Fig. 7). We also show that zinc is required for nitric oxide synthase activity, but the elevation of cytoplasmic zinc does not further increase the activity of the enzyme.

Cytoplasmic zinc is tightly regulated by 24 (ZIP and ZnT) transporters^23^. Currently, there are no agents that selectively interfere with the activity of zinc transporters, so in the present study we used a range of established zinc ionophores and chelators that bypass the complex regulation of zinc transport to investigate the roles of zinc in vascular physiology. All the zinc ionophores (with different chemical structures), but not extracellular zinc alone, showed similar vasorelaxant effects. Direct evidence for a role for zinc was obtained by administering zinc into SMC via a patch pipette. Furthermore, the contractile effects evoked by intracellular zinc chelation that occurred in certain vessels were neutralized by the addition of zinc. Using these approaches, we conclude that zinc, and not chemical scaffolds of the ionophores or chelators, induces changes to vascular tone.

To understand the mechanisms by which zinc causes vasorelaxation, we systematically explored the different primary vasorelaxant pathways in the different cells of the vasculature that may be altered by zinc, based on previous reports from other cell types or systems. While our results show that zinc ionophores have ranked potency sensory nerve > endothelium > smooth muscle for mediating vasorelaxation, it does not necessarily follow that this order indicates the relative importance of zinc signaling in each of these cells. Rather it may relate to the potency of these ionophores in delivering zinc according to cell type. For example, when zinc pyrithione was administered to isolated mesenteric arteries, it caused a CGRP-dependent relaxation at 0.3 µM but only suppressed the calcium-induced contraction at 10 µM in rat tissues, while it caused a CGRP-dependent relaxation at 3 µM in mouse tissues. However, when zinc was directly administered into the cytoplasm of SMCs from mice through a patch pipette, it potently suppressed VGCC currents at 1 nM, which is within the range of physiological levels of intracellular free zinc (ranging from hundreds of picomolar free basal level to ~1 nM during cellular transients or locally in microdomains^25^). The intracellular free zinc concentration that can be achieved by the zinc ionophores in each cell type will also depend on the buffering capacity and the type and abundance of zinc transporters expressed.

The relative potency of zinc modifying agents in different vascular beds may be impacted by the extent of vascular sensory innervation. In small mesenteric arteries, desensitization of the perivascular sensory nerves by capsaicin, inhibition of TRPA1 channels or blockade of CGRP receptors markedly decreased the potency of the zinc ionophores, confirming that zinc activates TRPA1 channels in perivascular sensory nerves to cause CGRP-dependent vasorelaxation. We found that in comparison to the mesenteric arteries, the larger vessels (including pulmonary, aorta and human vessels) were less sensitive to zinc delivery by zinc pyrithione and Zn(DTSM). This difference in sensitivity may relate to the dense perivascular CGRP innervation in mesenteric resistance vessels reported by us^42^ and others^43,44^. This suggestion is supported by our finding that zinc-bis-histidinate caused a CGRP-dependent increase in cutaneous blood flow in vivo in the rat hind paw, where the vasculature is also well innervated by sensory nerves. The role of zinc in sensory neurotransmission has been suggested previously based on the presence of zinc and its transporters in dorsal root ganglia neurons^45,46^, and zinc-mediated activation of TRPA1 channels in somatic sensory neurons^7,8^. This was further supported by evidence of decreased responsiveness to zinc pyrithione-induced vasodilation in TRPA1 KO mice in vitro and in vivo compared to the WT controls.

The potency of zinc pyrithione was also decreased by the removal of endothelium or by inhibition of cyclooxygenases with indomethacin suggesting a role for the endothelium in the vasorelaxant mechanism. Two prostanoids synthesized by cyclooxygenases in the endothelium or smooth muscle may be responsible for vasodilatory actions; prostacyclin (PGI_2_) that acts through prostanoid IP receptor^47^ and prostaglandin E2 that acts on EP_2_ and EP_4_ receptors expressed in SMCs^48,49^. Inhibition of prostacyclin synthesis was responsible for the effect shown by indomethacin because only the selective inhibition of the prostacyclin IP receptor, but not the prostanoid EP_2_ or EP_4_ receptors showed a similar effect. However, the decrease in the potency of zinc pyrithione caused by these treatments was much less than that with capsaicin pretreatment, demonstrating that sensory nerves are primarily responsible for the dilation to low concentrations of zinc pyrithione.

The voltage dependence of activation of the VGCC in the SMCs indicates that the current is mediated by L-type calcium channels. To our knowledge, there are no previous reports that intracellular zinc inhibits the activity of L-type channels in vascular smooth muscle, but there are reports that intracellular zinc inhibits L-type channels in cardiomyocytes^5,50^. As observed in the present study, the intracellular concentrations of zinc that inhibited L-type calcium currents in cardiomyocytes were in the nanomolar range. In cardiac myocytes, the decrease in the peak calcium current was associated with slowing of the time to peak current and of the time constant of inactivation^5^. By contrast, we observed that zinc caused no change in the voltage dependence of activation or the time constant of activation or inactivation for the VGCC. These findings suggest that intracellular zinc is acting as a pore blocker of L-type calcium channels.

Our results showed that cell-permeable zinc chelators fully contracted cerebral and coronary arteries in the rat, which contained low levels of zinc compared to the other vessels studied. Zinc chelators also caused depolarization and a decrease in membrane conductance of SMCs in rat mesenteric arteries, further confirming a physiological role of endogenous zinc. The contractile effect of the zinc chelators was also observed in the human saphenous vein, highlighting the potential importance of endogenous zinc in human vascular function. Our findings suggest that zinc is an important regulator of vascular tone and smooth muscle membrane potential and the differing actions of the zinc chelators on vascular tone may depend on the basal level of endogenous zinc in the vessel studied, but this possibility requires further investigation.

Zinc is reported to modulate the activity of many ion channels and enzymes that may be relevant in vascular physiology. Thus, we investigated the possibility of alternative mechanisms to explain its vasodilatory effects. Direct activation of potassium channels such as K_ATP_, BK_Ca_, and K_V_ by zinc is reported for other cell types, including pulmonary SMCs^6,9,39^, but we were unable to demonstrate a role for zinc indirectly affecting functionally relevant potassium channels in our experiments. Activation of K_ATP_ channels was important for the hyperpolarization caused by zinc ionophores in rat mesenteric arteries, but this action was mediated indirectly via the release of CGRP from the perivascular sensory nerves. However, inhibition of K_ATP_ channel by glibenclamide or of other potassium channels failed to change the vasorelaxant potency of zinc ionophores in rat mesenteric arteries contracted with U46619. This accords with previous reports that show glibenclamide blocks CGRP-induced hyperpolarization but fails to inhibit the CGRP-induced relaxation of rat mesenteric arteries contracted with U46619 or noradrenaline^32,40^. Therefore, CGRP can elicit relaxation via a mechanism that does not involve K_ATP_ channels. In support of this conclusion, Nelson et al.^33^ reported that complete blockade of K_ATP_ channel-induced hyperpolarization only decreased the relaxation to CGRP by ~50% in rabbit isolated mesenteric arteries contracted with noradrenaline. This finding indicates that in rabbit mesenteric arteries CGRP causes relaxation by at least two mechanisms, only one of which involves K_ATP_ channels. In rat mesenteric arteries, we assume that a vasorelaxant mechanism that does not involve K_ATP_ channels dominates under the conditions of our experiments.

It has also been reported that zinc is important for the activity of nitric oxide synthase, phosphodiesterase, and other mechanisms of relevance to vascular physiology^1–4^. However, neither inhibition of nitric oxide synthase nor of the downstream soluble guanylate cyclase impacted zinc ionophore-mediated vasorelaxation in our experiments. In contrast to the increase of cellular zinc by ionophores, the removal of endogenous “labile” zinc by using the zinc-selective chelator TPA caused a significantly decreased vasodilator potency of acetylcholine in isolated mesenteric arteries, where the mechanism of action depends on the activation of nitric oxide synthase. The vasodilatation produced by direct delivery of nitric oxide with sodium nitroprusside was not affected by zinc chelation, confirming that removal of endogenous zinc inhibited activation of nitric oxide synthase by acetylcholine. The importance of zinc in nitric oxide synthase function has previously been appreciated. For instance, Tomat et al.^19^ reported that moderate zinc deficiency during growth resulted in hypertension, by a mechanism that involved decreased nitric oxide synthase activity, while Kušleikaitė et al.^51^ showed that chronic zinc supplementation to rabbits under hypodynamic stress recovered acetylcholine-mediated relaxation responses in the thoracic aorta. These results underscore the importance of endogenous zinc for the activity of nitric oxide synthase, however, the addition of excess cytoplasmic zinc by using ionophores does not further increase its enzymatic activity.

Together with our findings demonstrate that elevating intracellular zinc causes vasorelaxation through its combined effects on TRPA1-mediated CGRP signaling from perivascular sensory nerves, endothelial synthesis of dilatory prostanoids, and inhibition of smooth muscle VGCCs. Thus, the vasodilatory mechanism(s) of zinc modifying agents in a particular vascular bed will be dictated by their differential regulation by sensory innervation, endothelial vasoactive agents, the VGCC subtypes and endogenous zinc levels. These factors are particularly important if zinc modulation is being considered in a therapeutic context, which may be advantageous when selectivity for certain vessels is desired. Therapeutic zinc-induced TRPA1 activation may be limited by side effects including pain. Therefore, determining the relative expression levels of the zinc transporters and their functional importance in zinc regulation in the different cell types and in different vascular beds and pathologies is now a priority for exploiting zinc biology for therapeutics. Understanding changes in cell-specific free zinc levels in disease and the vascular effects caused by modifying cytoplasmic zinc levels using delivery agents, chelators, or agents selective for zinc transporters may thus pave a new pathway for developing zinc-based treatments for cardio- and cerebrovascular diseases.

## Methods

### Ethics

All human protocols were performed in accordance with the World Medical Association Declaration of Helsinki—ethical principles for medical research involving human subjects (2018). The protocols and use of tissues were approved by the Institutional Review Board of TEDA International Cardiovascular Hospital (TICH, ethics approval no. [2016]-1219-2 and [2014]-1223-4) and informed consent was obtained from the patients or next of kin for the use of excess, discarded vessel segments. Human internal mammary artery^52^ and saphenous vein^53,54^ were collected during routine cardiac surgeries at TICH, Tianjin, China from 14 patients (8 males, 6 females) aged 55–75 years and immediately transported to the laboratory for experimentation within 5–15 min.

All animal experimental procedures were performed in accordance with the Australian Code for the care and use of animals for scientific purposes (8th edition, 2013, National Health and Medical Research Council, Canberra) and approved by the University of Melbourne Animal Ethics Committee (1212630, 1513798.1, and 1413363.1) or the University of Vermont Institutional Animal Care and Use Committee (protocol 18-004).

### Animals

Male Sprague Dawley rats (250–350 g) aged 8–9 weeks were obtained from the Biomedical Animal Facility, University of Melbourne, Victoria, Australia. Male C57BL/6J for patch–clamp experiments in isolated vascular SMCs, TRPA1 homozygous knockout (B6;129P-*Trpa1*^*tm1Kykw*^/J)^55^ (#006401) and B6129PF2/J wildtype mice (#100903) aged 8–12 weeks (25–30 g) were obtained from Jackson Laboratories, USA. All animals were group-housed in a climate-controlled facility (21 ± 1 °C) with ambient humidity, a 12-h dark/light cycle, and free access to food and water. Rats and TRPA1 KO and WT mice were deeply anesthetized by inhalation of 5% isoflurane (Baxter Healthcare, Australia) in O_2,_ and C57BL/6 J mice were anesthetized by intraperitoneal (i.p.) injection of sodium pentobarbital (100 mg/kg, Sigma, USA), and then they were euthanized by rapid decapitation.

### Functional in vitro protocols in isolated arteries

Human internal mammary artery and saphenous vein or a range of vessels from rats and mice (left main or anterior descending coronary arteries [250–400 µm internal diameter, i.d.], second-order pulmonary interlobar arteries [300–600 µm i.d.], second or third-order mesenteric arteries [200–350 µm i.d.], interlobar renal arteries [250–350 µm i.d.], saphenous arteries or veins [300–600 µm i.d.]), or the thoracic aorta was dissected and placed in ice-cold PSS-A with the following composition (mmol/L, mM): NaCl 119; KCl 4.69; MgSO_4_·7H_2_O 1.17; KH_2_PO_4_ 1.18; glucose 5.5; NaHCO_3_ 25; CaCl_2_·6H_2_O 2.5 saturated with carbogen (O_2_ 95%; CO_2_ 5%) at pH 7.4. In addition, rat middle cerebral (250–400 µm i.d.) and basilar (250–450 µm i.d.) arteries were used and for these vessels, the PSS-A contained 1.5 mM CaCl_2_ to minimize the occurrence of spontaneous contractions.

After isolation of vessels, ~2 mm length segments of arteries were mounted in myograph chambers (Model 610 M and 620 M; Danish Myo Technology, Denmark) containing PSS-A at 37 °C for isometric force measurement^54,56^. Contractile and relaxation responses using rat and mice isolated vessels were recorded with LabChart 7 and a PowerLab 4/30 A/D converter (AD Instruments Pty Ltd, Australia) while those including human isolated vessels were recorded with LabChart 8 (AD Instruments) and Myodaq and Myodata 2.01 (Maastricht University, Maastricht, Netherlands). To normalize the basal conditions, the vessels were passively stretched according to a normalization protocol and adjusted to a diameter setting of 90% of that determined for an equivalent transmural pressure of 100 mmHg (30 mmHg for veins). After allowing the tissues to equilibrate for 30 min, the arteries were exposed to a potassium depolarizing solution (124 mM K^+^ replacing Na^+^ in PSS; termed KPSS) and noradrenaline (10 µM) for 2 min (chemicals, their source and preparation are provided in the online supplement). A second exposure to KPSS solution (only) was used to provide a reference contraction.

For the zinc chelators TPA (10–300 µM) or TPEN (30–300 µM), cumulative concentration responses were constructed. For relaxation experiments, an initial stable contraction was obtained using U46619, endothelin 1 or PSS containing 62 mM K^+^ (equimolar substitution of K^+^ for Na^+^) before completing concentration–response curves to acetylcholine (0.001–3 µM) or the different zinc ionophores in the absence or presence of a pretreatment. The concentration of the contractile agent was adjusted to produce a stable contraction that was about 80% of that produced by KPSS. To test the effects of zinc ionophores in VGCC-mediated contraction, tissues were washed with a “calcium-free” buffer followed by the addition of high potassium solution (80 mM KCl) to cause depolarization-dependent opening of VGCC. Then increasing concentrations of calcium (0.01–10 mM) were added to the bath to cause maximum contraction (control first curve) followed by the presence of different concentrations of a zinc ionophore (second curve). Arteries were incubated with a single concentration of a zinc ionophore for 15–20 min before the second concentration–contraction curve for calcium was completed.

To remove the effects of activating peptidergic perivascular sensory nerves, a subset of the arteries was incubated with capsaicin 10 µM for 30 min followed by washout to prevent non-specific actions of capsaicin. To investigate the role of the endothelium it was removed by gently rubbing the lumen using human hair. Only arteries with a >90% decrease in the relaxation to acetylcholine and no change in the contraction to potassium depolarizing solution (124 mM K^+^ replacing Na^+^ in PSS; termed KPSS) were used in the experiments.

### In vivo blood pressure and flow measurement

Rats and mice were anesthetized by inhalation of 5% isoflurane (in O_2_) followed by i.p. injection of pentobarbitone in rats (60 mg/kg; TROY Laboratories, NSW, Australia, maintained by top-up doses of 10–20 mg/kg i.v.) or i.p. injection of urethane (2.5 g/kg; Sigma, maintained by top-up doses of 0.05–0.1 g/kg i.v.). Animals were placed on a homeostatic blanket system to maintain body temperature at 37 ± 1 °C. Lignocaine (1% s.c.) was injected for placement of a tracheotomy tube (for mechanical ventilation), carotid artery, and jugular vein catheters. Atropine (1 mg/kg s.c.) was administered to inhibit bronchial secretions.

All drug treatments were administered via the jugular vein catheter. Pulsatile blood pressure, mean arterial pressure (MAP) and heart rate (HR; beats/min) were measured via the carotid artery catheter filled with heparinized (10 U/ml; Pfizer, NY, USA) saline 0.9% and connected to a pressure transducer (Argon Medical Devices, Athens, Greece) and the output was recorded with LabChart 7 and a Powerlab 8SP (AD Instruments). A Silastic-cuffed Doppler flow probe placed around the aorta just above the bifurcation was used for the constant recording of hindquarter blood flow (kHz) and derivation of hindquarter vascular conductance (kHz/MAP mmHg).

The animal was allowed to stabilize for 30 min to establish baseline parameters. A single dose of Zn(DTSM), 1 mg/kg, or zinc pyrithione, 0.3 mg/kg, was then administered i.v. and haemodynamic measurements were recorded continuously for 10 min or until the MAP returned to the baseline level. Then, the effects of increasing non-cumulative i.v. doses of Zn(DTSM) (3, 10, and 30 mg/kg), zinc pyrithione (1 and 3 mg/kg), or an equivalent volume of the vehicle to that used for each dose was tested.

For the measurement of sensory nerve-mediated vasodilation by zinc-bis(histidinate), the rat hind paw, which is well innervated by sympathetic and sensory nerves^57^, was used. Rats were anesthetized with urethane (2 g/kg i.p.) after light anesthesia with inhalation of 5% isoflurane. A laser Doppler probe (OxyFlo probe MSP300XP; ADI Blood Flowmeter) was placed on one hind paw (hair removed with depilatory cream). The animal was allowed to stabilize for 30 min to establish baseline parameters. A single i.v. dose of zinc-bis(histidinate) was then administered at 3 mg/kg. The hemodynamic and cutaneous blood flow measurements were recorded continuously for 15 min. Then, the effects of 10 and 30 mg/kg doses of zinc-bis(histidinate) were tested. To assess the contribution of CGRP released from sensory nerves, the receptor antagonist BIBN4096 (3 mg/kg, a dose reported to block sensory nerve-mediated vasodilatation^35^) was administered i.v. by slow infusion over 10 min before testing the effects of zinc-bis(histidinate).

### Electrophysiological measurements in intact arteries

Segments of rat second-order mesenteric artery were pinned to a Sylgard-coated base of a recording chamber and continuously perfused (4 ml/min) with PSS-B of the following composition (mM): NaCl 133.4; KCl 4.7; MgCl_2_ 1.2; KH_2_PO_4_ 1.3; glucose 7.8; NaHCO_3_ 16.3; CaCl_2_ 2.0 saturated with carbogen (O_2_ 95%; CO_2_ 5%; to pH 7.3) containing the α_1_-adrenoceptor antagonist prazosin (0.1 μM, to decrease the likelihood of contraction dislodging the recording electrode) and maintained at 36–37 °C. The proximal end of the mesenteric artery was cleared of fat and drawn into a suction stimulating electrode and the perivascular nerves excited electrically (1 ms pulse width, 10 V). Electrophysiological recordings were made at a site 3–5 mm distal of the mouth of the suction stimulating electrode. At this site, the connective tissue and fat cells were carefully removed to reveal the surface of the artery. Intracellular recordings were made from SMCs using borosilicate glass microelectrodes (100–150 MΩ) filled with 0.5 M KCl and connected to Axoclamp bridge amplifier (Axon Instruments Inc., CA, USA). Membrane potentials were determined upon withdrawal of the microelectrode. Impalements were only accepted if (1) the cell penetration was abrupt, (2) the membrane potential increased to a value more negative than the initial potential, and (3) the membrane potential was stable.

The effects of chemical agents on membrane potential and the purinergic EJPs evoked by trains of five stimuli at 1 Hz (delivered via a suction electrode applied to the proximal of the vessel segment) were determined in single-cell experiments in which both control and test recordings were made during the same impalement. To remove effects mediated through the perivascular sensory nerves, the tissues were pretreated with 10 µM capsaicin for 10 min, and following washout, they were left for at least 20 min before starting the experiment. Recordings from three or four cells under control conditions were made before testing the effects of chemical agents.

In vascular smooth muscle, the EJP time constant of decay (τEJP) is similar to the membrane time constant; changes in τEJP produced by applied agents most likely indicate a change in membrane resistance^58^. The membrane time constant is the product of membrane resistance and capacitance and it is unlikely that membrane capacitance is changed by the applied agents. The τEJP was estimated by fitting a mono-exponential function to the decay phase of the EJP using the in-built functions of Igor Pro (Wavemetrics, OR, USA). The relative change in membrane conductance produced by an applied agent was estimated by dividing the τEJP measured just before its application by that measured in its presence.

### Electrophysiological recordings in isolated SMCs

SMCs from mesenteric arteries of mice were isolated enzymatically^59,60^. Arterial segments were placed in isolation solution (mM): NaCl 60, Na^+^ glutamate 80, KCl 5, MgCl_2_ 2, glucose 10, and HEPES 10, pH 7.4 followed by a two-step enzymatic digestion protocol. (1) a 13–15 min incubation (37 °C) in isolation solution containing 0.5 mg/ml papain and 1.5 mg/ml dithioerythritol followed by (2) a 10 min incubation in isolation solution containing 0.65 mg/ml type-F collagenase, 0.35 mg/ml type-H collagenase and 100 µM Ca^2+^ (37 °C). Tissues were then washed with ice-cold isolation solution and triturated with a fire-polished pipette. Cells were kept in ice-cold isolation solution (4 °C) after trituration for use within the same day within ~6 h. Cells were left (~30 min) to settle down to the coverslip of the experimental chamber before recordings.

Conventional patch–clamp electrophysiology was used to monitor whole-cell currents in the SMCs^59^. Patch pipettes (~4 MΩ) pulled from borosilicate glass (Sutter Instruments, USA) were covered with wax and filled with pipette solution of the following composition (mM): CsCl 135, MgCl_2_ 1, Mg-ATP 2, HEPES 10, EGTA 10, pH 7.2 (CsOH). To test the effects of intracellular Zn^2+^, ZnCl_2_ was included in the pipette solution to a final free concentration of zinc of 1 or 10 nM calculated using Maxchelator software (https://somapp.ucdmc.ucdavis.edu/pharmacology/bers/maxchelator/webmaxc/webmaxcS.htm, ionic strength 0.15, 22 °C, pH 7.2). Bath solution composition was (mM): NaCl 110, CsCl 1, BaCl_2_ 10, MgCl_2_ 1.2, glucose 10 and HEPES 10, pH 7.4. After gaining electrical access, cells were left to stabilize (~3 min after gaining electrical access) before recordings to ensure adequate intracellular dialysis. For ionophore experiments, SMCs were preincubated with 10 µM zinc pyrithione for at least 10 min before recording.

Whole-cell currents were recorded using an Axopatch 200B patch–clamp amplifier (Axon Instruments, USA), filtered at 1 kHz, digitized at 5 kHz, and stored on a computer for analysis using Clampfit 10.3. Whole-cell capacitance ranged between 12–21 pF and access resistance averaged around 5–10 MΩ. SMCs were voltage-clamped at a holding potential of −60 mV. For ramp protocols, a voltage ramp from −90 to +40 mV (0.45 mV/ms) was used. For step protocols, a −90 mV pre-pulse (200 ms) followed by 13 voltage steps (300 ms) ranging from −70 to +50 mV was used. Current–voltage relationships were plotted as peak current density (pA/pF) at each voltage step. Activation and inactivation time constants (*τ*) were calculated by the exponential fitting of the activating or inactivating segments of the VGCC currents, respectively. Voltage dependence of activation was assessed by a step protocol and currents elicited by the test voltage were normalized to maximal current to plot %I/Imax against the voltage step. The voltage dependence of activation was assessed using single Boltzmann distribution of the following form (1):

1 $${\rm{Activation}}:I(V)=\frac{{I}_{{{\max }}}}{1+\exp [\frac{V-{V}_{50}}{k}]}$$

where *I*_max_ is the maximal activatable current, *V*_50_ is the voltage at which the current is 50% activated or inactivated, and *k* is the slope (voltage-dependence) of the distribution.

### Intracellular sodium measurements using hTRPA1 or hTRPV1 transfected HEK cells

T-Rex-293 Human embryonic kidney 293 (HEK293) FlpIn cells (Invitrogen, Australia) stably expressing human TRPA1 or TRPV1 that have been previously described and characterized^61^ were used. Cells were maintained in DMEM supplemented with 10% fetal bovine serum, penicillin (100 U/ml), streptomycin (100 µg/ml), and hygromycin (200 µg/ml) and plated in poly-d-lysin coated 96-well plates at an approximate density of 40,000 cells per well. Cells were grown for 24 h and expression of hTRPA1 or hTRPV1 was induced by adding 1 µg/ml tetracycline (Sigma) for 4 h. Cells were then washed with HEPES buffer (mM): NaCl 140; KCl 5; MgCl_2_ 1.0; CaCl_2_ 2.0; HEPES 10; glucose 11; pH 7.4 and loaded with 5 µM SBFI-AM (Santa Cruz Biotechnology, USA) in the presence of 1 mM probenecid and 0.01% Pluronic F-127 for 1 h at 37 °C. Fluorescence emission ratios were collected at 505 nm after excitation at 340 and 380 nm wavelengths. Zinc pyrithione (0.1–3 µM) or positive controls—cinnamaldehyde (300 µM; TRPA1 agonist), capsaicin (3 µM; TRPV1 agonist) or Triton-X (0.1%; increases sodium uptake by membrane permeabilization) – were applied to cells at approximately 30 s into the recording by a Flexstation 3 Multi-Mode microplate reader (Molecular Devices, USA). The mean of the peak fluorescence ratio change from baseline (for three wells per compound per concentration) was used to plot concentration-response curves.

### Data analyses

All data are expressed as mean ± SEM, with n being the number of rats/mice/humans or arteries isolated from separate animals unless stated otherwise. Each sigmoidal concentration-response curve was fitted using Prism 8 (GraphPad Software, USA). In some cases, the last data point (i.e., highest concentration) was imputed (replication of the next highest concentration) for better sigmoidal fitting. The *p*EC_50_ values (the negative log_10_M of drug concentration that decreases the response by 50%) and *E*_max_ (maximum response) were determined for each tissue and averaged.

Two-tailed Student’s paired and unpaired *t* test were used to analyze the differences between two variables and one-way analysis of variance (1-way ANOVA) with Dunnett’s post-test was used to compare means between three or more variables. Where the variances were significantly different (assessed by the Brown–Forsythe test), Brown-Forsythe Welch 1-way ANOVA with Dunnett T3 post-test or the non-parametric Kruskal-Wallis test with Dunn’s post-test were used instead of 1-way ANOVA. Repeated measures one-way ANOVA (mixed-effects if there were missing values) with Dunnett’s post-test was used to test concentration-dependent changes from baseline values within the same group. The *p* values from the post-tests are reported. For in vivo experiments and comparison of two or more concentration-response curves, repeated measures (mixed-effects if there were missing values) two-way analysis of variance (two-way ANOVA) with Dunnett’s post-test for multiple comparisons of treatment, time or dose were used. The adjusted *p* values after individual comparisons (post-test) are reported. Values of *p* < 0.05 were considered statistically significant.

For in vivo hemodynamic parameters in anesthetized rats and mice, the average of the values at 5 min before (−5) and immediately before addition (0 min) of the first dose of the test drug was used as a baseline. The values at 2, 3, 4, 5, and 10 min after administration of a single dose of the test drug or vehicle equivalent were measured as well as the change from baseline values in MAP at the 5 min (close to the peak effect) and the area under the curve (AUC) for the change from the baseline values (Prism 8 software). In one rat experiment, Zn(DTSM) at the highest dose was lethal and only the data for the previous doses were used. In one WT mouse, the highest dose of zinc pyrithione was lethal after 5 min recording, hence, the last reasonable data were used for analysis.

For in vivo measurements of cutaneous blood flow, an average of 3 s consecutive values calculated every 5 s were computed by LabChart software, and the data were transformed as a percentage of baseline (an average of 1.5 min recording before each dose). The AUC for change from baseline values was calculated in Prism 8 software. MAP, hindquarter flow, and conductance were taken immediately before and every 5 min after a single dose of treatment.

### Reporting summary

Further information on research design is available in the Nature Research Reporting Summary linked to this article.

## Supplementary information

Supplementary Information

Reporting Summary

## Data Availability

The data supporting the main findings of this study are included in the paper and the supplementary file. All other data used in this study are available on request. Source data are provided with this paper.

## Source data

Source Data

## References

[1] von Bulow V, Rink L, Haase H (2005). Zinc-mediated inhibition of cyclic nucleotide phosphodiesterase activity and expression suppresses TNF-α and IL-1β production in monocytes by elevation of guanosine 3′,5′-cyclic monophosphate. J. Immunol..

[2] Chreifi G (2014). Communication between the zinc and tetrahydrobiopterin binding sites in nitric oxide synthase. Biochemistry.

[3] Bunning P, Riordan JF (1985). The functional role of zinc in angiotensin converting enzyme: implications for the enzyme mechanism. J. Inorg. Biochem..

[4] Roques BP, Noble F, Dauge V, Fournie-Zaluski MC, Beaumont A (1993). Neutral endopeptidase 24.11: structure, inhibition, and experimental and clinical pharmacology. Pharm. Rev..

[5] Alvarez-Collazo J, Diaz-Garcia CM, Lopez-Medina AI, Vassort G, Alvarez JL (2012). Zinc modulation of basal and β-adrenergically stimulated L-type Ca^2+^ current in rat ventricular cardiomyocytes: consequences in cardiac diseases. Pflug. Arch..

[6] Xiong Q, Sun H, Li M (2007). Zinc pyrithione-mediated activation of voltage-gated KCNQ potassium channels rescues epileptogenic mutants. Nat. Chem. Biol..

[7] Andersson DA, Gentry C, Moss S, Bevan S (2009). Clioquinol and pyrithione activate TRPA1 by increasing intracellular Zn^2+^. Proc. Natl Acad. Sci. USA.

[8] Hu H, Bandell M, Petrus MJ, Zhu MX, Patapoutian A (2009). Zinc activates damage-sensing TRPA1 ion channels. Nat. Chem. Biol..

[9] Prost AL, Bloc A, Hussy N, Derand R, Vivaudou M (2004). Zinc is both an intracellular and extracellular regulator of K_ATP_ channel function. J. Physiol..

[10] Luo J (2018). Zinc inhibits TRPV1 to alleviate chemotherapy-induced neuropathic pain. J. Neurosci..

[11] Toth A (2014). Vanilloid receptor-1 (TRPV1) expression and function in the vasculature of the rat. J. Histochem. Cytochem..

[12] Pozsgai G (2012). The role of transient receptor potential ankyrin 1 (TRPA1) receptor activation in hydrogen-sulphide-induced CGRP-release and vasodilation. Eur. J. Pharm..

[13] Eberhardt M (2014). H_2_S and NO cooperatively regulate vascular tone by activating a neuroendocrine HNO-TRPA1-CGRP signalling pathway. Nat. Commun..

[14] Bautista DM (2005). Pungent products from garlic activate the sensory ion channel TRPA1. Proc. Natl Acad. Sci. USA.

[15] Hansted AK, Bhatt DK, Olesen J, Jensen LJ, Jansen-Olesen I (2019). Effect of TRPA1 activator allyl isothiocyanate (AITC) on rat dural and pial arteries. Pharm. Rep..

[16] Pozsgai G (2010). Evidence for the pathophysiological relevance of TRPA1 receptors in the cardiovascular system in vivo. Cardiovasc. Res..

[17] Mousavi SM (2020). The effect of zinc supplementation on blood pressure: a systematic review and dose–response meta-analysis of randomized-controlled trials. Eur. J. Nutr..

[18] Williams CR (2019). Zinc deficiency induces hypertension by promoting renal Na^+^ reabsorption. Am. J. Physiol..

[19] Tomat AL (2005). Moderate zinc deficiency influences arterial blood pressure and vascular nitric oxide pathway in growing rats. Pediatr. Res..

[20] Kunutsor SK, Laukkanen JA (2016). Serum zinc concentrations and incident hypertension: new findings from a population-based cohort study. J. Hypertens..

[21] Bergomi M (1997). Zinc and copper status and blood pressure. J. Trace Elem. Med. Biol..

[22] Ladefoged K, Hagen K (1988). Correlation between concentrations of magnesium, zinc, and potassium in plasma, erythrocytes and muscles. Clin. Chim. Acta.

[23] Kambe T, Tsuji T, Hashimoto A, Itsumura N (2015). The physiological, biochemical, and molecular roles of zinc transporters in zinc homeostasis and metabolism. Physiol. Rev..

[24] Garwin SA (2019). Interrogating intracellular zinc chemistry with a long stokes shift zinc probe ZincBY-4. J. Am. Chem. Soc..

[25] Maret W (2017). Zinc in cellular regulation: the nature and significance of “zinc signals”. Int. J. Mol. Sci..

[26] Zhao L (2015). The zinc transporter ZIP12 regulates the pulmonary vascular response to chronic hypoxia. Nature.

[27] Perez Y (2017). SLC30A9 mutation affecting intracellular zinc homeostasis causes a novel cerebro-renal syndrome. Brain.

[28] Ehret GB (2016). The genetics of blood pressure regulation and its target organs from association studies in 342,415 individuals. Nat. Genet..

[29] International Consortium for Blood Pressure Genome-Wide Association S. (2011). Genetic variants in novel pathways influence blood pressure and cardiovascular disease risk. Nature.

[30] Robertson PA (1960). Calcium and contractility in depolarized smooth Muscle. Nature.

[31] Prandi F, Jimenez-Vargas J (1954). Influence of potassium on contractility of bronchial muscle. Rev. Esp. Fisiol..

[32] Dunn WR, Hardy TA, Brock JA (2003). Electrophysiological effects of activating the peptidergic primary afferent innervation of rat mesenteric arteries. Br. J. Pharm..

[33] Nelson MT, Huang Y, Brayden JE, Hescheler J, Standen NB (1990). Arterial dilations in response to calcitonin gene-related peptide involve activation of K^+^ channels. Nature.

[34] Kirillova-Woytke I, Baron R, Jänig W (2014). Reflex inhibition of cutaneous and muscle vasoconstrictor neurons during stimulation of cutaneous and muscle nociceptors. J. Neurophysiol..

[35] Aviles-Rosas VH (2017). Olcegepant blocks neurogenic and non-neurogenic CGRPergic vasodepressor responses and facilitates noradrenergic vasopressor responses in pithed rats. Br. J. Pharm..

[36] Earley S, Brayden JE (2015). Transient receptor potential channels in the vasculature. Physiol. Rev..

[37] Zygmunt PM, Hogestatt ED (2014). TRPA1. Handb. Exp. Pharm..

[38] Figueroa JA, Vignesh KS, Deepe GS, Caruso J (2014). Selectivity and specificity of small molecule fluorescent dyes/probes used for the detection of Zn^2+^ and Ca^2+^ in cells. Metallomics.

[39] Eid BG, Gurney AM (2018). Zinc pyrithione activates K^+^ channels and hyperpolarizes the membrane of rat pulmonary artery smooth muscle cells. PLoS ONE.

[40] Lei S, Mulvany MJ, Nyborg NC (1994). Characterization of the CGRP receptor and mechanisms of action in rat mesenteric small arteries. Pharm. Toxicol..

[41] Park SJ, Min SH, Kang HW, Lee JH (2015). Differential zinc permeation and blockade of L-type Ca^2+^ channel isoforms Cav1.2 and Cav1.3. Biochim. Biophys. Acta.

[42] Johansen NJ, Tripovic D, Brock JA (2013). Streptozotocin-induced diabetes differentially affects sympathetic innervation and control of plantar metatarsal and mesenteric arteries in the rat. Am. J. Physiol. Heart Circ. Physiol..

[43] Kawasaki H, Takasaki K, Saito A, Goto K (1988). Calcitonin gene-related peptide acts as a novel vasodilator neurotransmitter in mesenteric resistance vessels of the rat. Nature.

[44] Yokomizo A, Takatori S, Hashikawa-Hobara N, Goda M, Kawasaki H (2015). Characterization of perivascular nerve distribution in rat mesenteric small arteries. Biol. Pharm. Bull..

[45] Zhang L (2007). Imunoreactivity of zinc transporter 7 (ZNT7) in mouse dorsal root ganglia. Brain Res. Bull..

[46] Velázquez RA, Cai Y, Shi Q, Larson AA (1999). The distribution of zinc selenite and expression of metallothionein-III mRNA in the spinal cord and dorsal root ganglia of the rat suggest a role for zinc in sensory transmission. J. Neurosci..

[47] Bley KR (2006). RO1138452 and RO3244794: characterization of structurally distinct, potent and selective IP (prostacyclin) receptor antagonists. Br. J. Pharm..

[48] af Forselles KJ (2011). In vitro and in vivo characterization of PF-04418948, a novel, potent and selective prostaglandin EP_2_ receptor antagonist. Br. J. Pharm..

[49] Foudi N (2008). Vasorelaxation induced by prostaglandin E2 in human pulmonary vein: role of the EP4 receptor subtype. Br. J. Pharm..

[50] Turan B (2003). Zinc-induced changes in ionic currents of cardiomyocytes. Biol. Trace Elem. Res.

[51] Kusleikaite M, Stonkus S, Laukeviciene A, Kusleika G (2003). The significance of zinc for contractility of smooth muscles and ultrastructure of their microfilaments in case of hypodynamic stress. Medicines.

[52] He GW (1992). Inhibitory effects of glyceryl trinitrate on alpha-adrenoceptor mediated contraction in the human internal mammary artery. Br. J. Clin. Pharm..

[53] He GW, Rosenfeldt FL, Angus JA (1993). Pharmacological relaxation of the saphenous vein during harvesting for coronary artery bypass grafting. Ann. Thorac. Surg..

[54] Angus JA, Wright CE (2000). Techniques to study the pharmacodynamics of isolated large and small blood vessels. J. Pharm. Toxicol. Methods.

[55] Kwan KY (2006). TRPA1 contributes to cold, mechanical, and chemical nociception but is not essential for hair-cell transduction. Neuron.

[56] Betrie AH (2017). Evidence of a cardiovascular function for microtubule-associated protein tau. J. Alzheimers Dis..

[57] Ruocco I, Cuello AC, Parent A, Ribeiro-da-Silva A (2002). Skin blood vessels are simultaneously innervated by sensory, sympathetic, and parasympathetic fibers. J. Comp. Neurol..

[58] Cassell JF, McLachlan EM, Sittiracha T (1988). The effect of temperature on neuromuscular transmission in the main caudal artery of the rat. J. Physiol..

[59] Harraz OF, Welsh DG (2013). Protein kinase A regulation of T-type Ca^2+^ channels in rat cerebral arterial smooth muscle. J. Cell Sci..

[60] Porter VA (1998). Frequency modulation of Ca^2+^ sparks is involved in regulation of arterial diameter by cyclic nucleotides. Am. J. Physiol..

[61] Lieu T (2014). The bile acid receptor TGR5 activates the TRPA1 channel to induce itch in mice. Gastroenterology.

