# Supplementary material for: Zinc drives vasorelaxation by acting in sensory nerves, endothelium and smooth muscle
Source: Nat Commun. 2021 Jun 1;12:3296. doi: 10.1038/s41467-021-23198-6 (PMC8169932; doi:10.1038/s41467-021-23198-6)
Supplement: Supplementary file 1 — Supplementary Information [file 41467_2021_23198_MOESM1_ESM.pdf]

Supplementary Materials for

Zinc drives vasorelaxation by acting in sensory nerves, endothelium and smooth muscle

Authors: Ashenafi H. Betrie[1,2,3], James A. Brock[4], Osama F. Harraz[5,6], Ashley I. Bush[1], Guo-Wei He[3], Mark T. Nelson[5,6,7], James A. Angus[2], Christine E. Wright[2*], Scott Ayton[1*]

Correspondence to:

Christine E. Wright, Cardiovascular Therapeutics Unit, Department of Biochemistry and Pharmacology, The University of Melbourne, VIC 3010, Australia. Tel.: +61 3 8344 8219; E-mail: cewright@unimelb.edu.au

and

Scott Ayton, Florey Institute of Neuroscience and Mental Health, The University of Melbourne, VIC 3052, Australia. Tel.: +61 3 9035 6559; Email: scott.ayton@florey.edu.au

This file includes:

Supplementary methods

Supplementary Fig. 1 to 13

Supplementary Table 1 to 3

Supplementary References

Supplementary methods

Electrical field stimulation of perivascular sympathetic nerves

Prior to investigating the effects of zinc ionophores on sympathetic nerve-evoked contractions of small mesenteric arteries, the tissues were incubated with prazosin (0.1 μmol/L, to protect the α_1-adrenoceptors) followed 5 min later by benextramine (3 μmol/L) for 5 min to produce irreversible blockade of prejunctional α_2-adrenoceptors[1]. Following extensive washing, it was confirmed that arteries contracted to noradrenaline (10 μmol/L). The tissues were then electrically stimulated with three 3 s trains at 25 Hz (0.25 ms, 30 V) at 1 min intervals to obtain control baseline contractions (the third response was used as a reference to normalize the responses). After 15 min, a frequency-response curve (4, 8, 16 and 32 Hz) was completed. A single concentration of the ionophore was then added for 30 min before repeating the frequency-response curve. In each experiment, it was confirmed that the electrically evoked contractions were fully blocked by tetrodotoxin (0.1 μmol/L), confirming they were due to neuronal stimulation.

Metal measurements using Inductively Coupled Plasma Mass Spectrometry

Metal levels from isolated tissues were measured using Inductively Coupled Plasma Mass Spectrometry (ICP-MS; Agilent 7700 series, Agilent Technologies, Santa Clara, CA, USA) under routine multi-element operating conditions using a Helium reaction gas cell, as previously described[2]. Tissue samples were weighed, freeze-dried, and then resuspended in 69% nitric acid (ultraclean grade, Aristar) overnight. The samples were then heated for 20 min at 90˚C, and equivalent volume of hydrogen peroxide (30%, Merck) was added for further 15 min incubation at 70˚C. The samples were diluted in double-distilled water and assayed by ICP-MS. Each tissue sample was measured in triplicate and the concentrations determined from the standard curve were normalized to wet tissue weight.

Chemicals

The chemicals used and suppliers were: acetylcholine bromide (Sigma, St. Louis, MO, USA), apamin (AusPep, Parkville, Victoria, Australia), 4-aminopyridine (Sigma), AM 0902 (1-[[3-[2-(4-Chlorophenyl)ethyl]-1,2,4-oxadiazol-5-yl]methyl]-1,7-dihydro-7-methyl-6H-purin-6-one; Tocris Biosciences, Bristol, UK), arginine vasopressin (AusPep), benextramine tetrachloride (Sigma), calcium chloride solution (Scharlab, Sentmenat, Spain), BIBN4096 (olcegepant; 1-[3,5-Dibromo-N-[[4-(1,4-dihydro-2-oxo-3(2H)-quinazolinyl)-1-piperidinyl]carbonyl]-D-tyrosyl-L-lysyl]-4-(4-pyridinyl)-piperazine; Tocris), capsaicin (Sigma), capsazepine (Sigma) rCGRP (AusPep), CAY10441 (RO1138452, 4,5-dihydro-N-[4-[[4-(1-methylethoxy)phenyl]methyl]phenyl]-1H-imadazol-2-amine, Cayman Chemicals, Ann Arbor, MI, USA) charybdotoxin (Synpeptide, Shanghai, China), clioquinol (5-chloro-7-iodoquinolin-8-ol) (gifts from Warner Babcock Institute for Green Chemistry, Massachusetts, USA), disulfiram (bis(diethylthiocarbamyl) disulfide, Sigma), endothelin-1 (AusPep), glibenclamide (Sigma), HC030031 (2-(1,3-Dimethyl-2,6-dioxo-1,2,3,6-tetrahydro-7H-purin-7-yl)-N-(4-isopropylphenyl)acetamide; Tocris), indomethacin (Sigma), [–]-noradrenaline bitartrate (Sigma), L-histidine (Sigma), L-NAME (N^{ω}-nitro-L-arginine-methyl ester hydrochloride; Sigma), L-161982 (N-[[4'-[[3-butyl-1,5-dihydro-5-oxo-1-[2-(trifluoromethyl)phenyl]-4H-1,2,4-triazol-4-yl]methyl][1,1'-biphenyl]-2-yl]sulfonyl]-3-methyl-2-thiophenecarboxamide, Cayman), PF-04418948 (1-(4-fluorobenzoyl)-3-[[(6-methoxy-2-naphthalenyl)oxy]methyl]-3-azetidinecarboxylic acid, Cayman), potassium chloride (Chem-Supply, South Australia, Australia), ODQ (1H-[1,2,4]Oxadiazolo[4,3-a]quinoxalin-1-one, Sigma), prazosin hydrochloride (Sigma), pyrithione (1-hydroxypyridine-2-thione sodium salt; Sigma), ruthenium red (Sigma), tetrodotoxin (Sigma), sildenafil (Sigma), TPA (Tris(2-pyridylmethyl)amine, Sigma), TPEN (N,N,N’,N’-*tetrakis*(2-pyridylmethyl)ethylenediamine,

Sigma), U46619 (9,11-dideoxy-9a,11a-methanoepoxy prostaglandin $F_{2\alpha}$; Tocris), zinc chloride (Sigma) and Zn(DTSM) (Zinc(II)3,4-hexanedione bis[N(4)-methylthiosemicarbazone]) (gifts from Warner Babcock Institute for Green Chemistry).

Stock solutions of 10 mM were made in DMSO (Ajax Finechem, Taren Point, NSW, Australia) for clioquinol, Zn(DTSM), BIBN4096 and U46619. Ruthenium red was made to 10 mM in ultra-pure water. Stock solutions of TPEN in in ethanol or TPA and HC030031 in DMSO were prepared at 100 mM. Subsequent serial dilutions were made in ultra-pure water (Milli-Q, Merck Millipore), except for clioquinol and disulfiram which were diluted to 1 mM in DMSO before being diluted in ultrapure water. All other drugs were prepared as stock solutions in Milli-Q water at 10 mM. Stock solutions were stored at -20°C except for pyrithione and disulfiram which were made daily to 20 mM in DMSO without or with addition of an equal volumes of 20 mM zinc chloride (Sigma) to a final concentration of 10 mM.

For *in vivo* experimental protocols, stock solution of 20 mg/ml were made fresh daily in 100% DMSO for Zn(DTSM) and diluted accordingly. For zinc-bis(histidinate) experiments, stock solutions of 20 mg/ml $ZnCl_2$ and 45 mg/ml L-histidine were made, and equal volumes mixed to give a 10 mg/ml zinc in a 1:2 stoichiometry with L-histidine. For BIBN4096, a stock solution of 1.8 mg/ml was made from a 10 mM solution in DMSO and further diluted in 20% (v/v) PEG400 in normal saline.

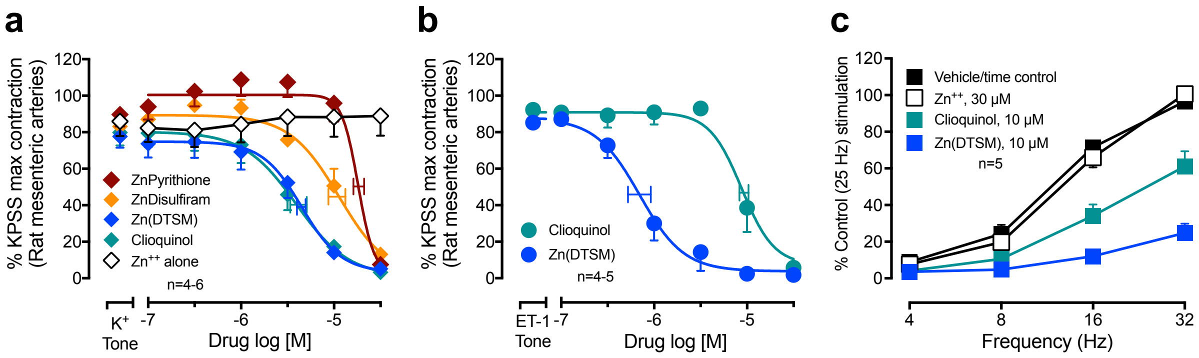

Supplementary Fig. 1: Relaxation induced by zinc ionophores in rat mesenteric arteries contracted with different agents. Concentration-relaxation curves for zinc ionophores in rat isolated mesenteric arteries contracted with (**a**) high potassium (62 mM in PSS-A, 9.6 ± 0.5 mN contraction, n=24) (K^+ Tone), or (**b**) endothelin-1 (ET-1 tone, 10 ± 1 mN contraction, n=9). Extracellular zinc (Zn^{++} alone) did not reduce K^+ Tone. Responses are expressed as % KPSS. (**C**) Stimulation frequency-contraction curves for electrical activation of perivascular sympathetic nerves (with 3 second trains) in the absence (vehicle/time control) or presence of a single concentration of zinc (Zn^{++} alone), clioquinol or Zn(DTSM). In **a** and **b**, the responses are expressed as % of the KPSS (124 mM K^+)- evoked contractions, whereas in **c** they are expressed as a % of the response to that to 25 Hz under control conditions. Vertical error bars are ± SEM and horizontal error bars are the average EC_{50} ± SEM. n, number of arteries isolated from separate rats.

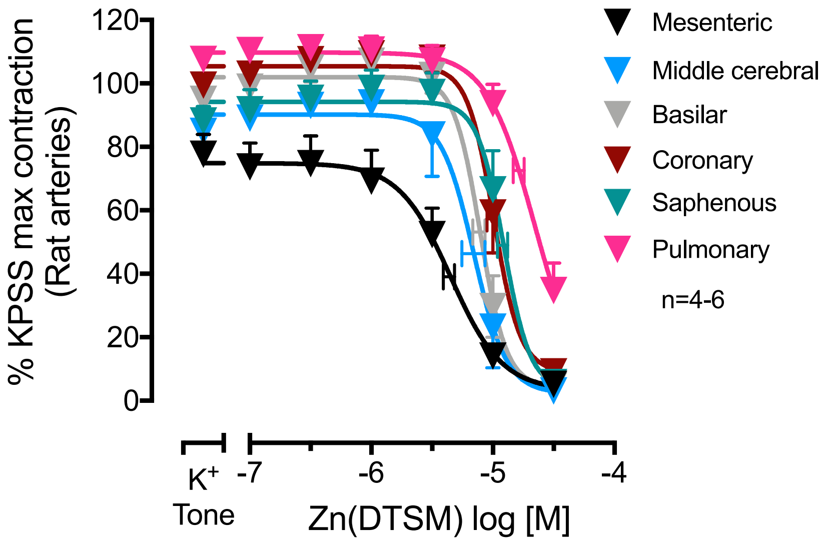

Supplementary Fig. 2: The zinc ionophore Zn(DTSM) relaxed arteries isolated from different vascular beds of rats. Zn(DTSM) relaxation of high potassium (62 mM in PSS-A) contracted mesenteric, middle cerebral, basilar, coronary, saphenous and pulmonary arteries. Data are expressed as % of the KPSS (124 mM K^+) reference contraction. Vertical error bars are ± SEM and horizontal error bars are the average EC_{50} ± SEM. n, number of arteries isolated from separate rats.

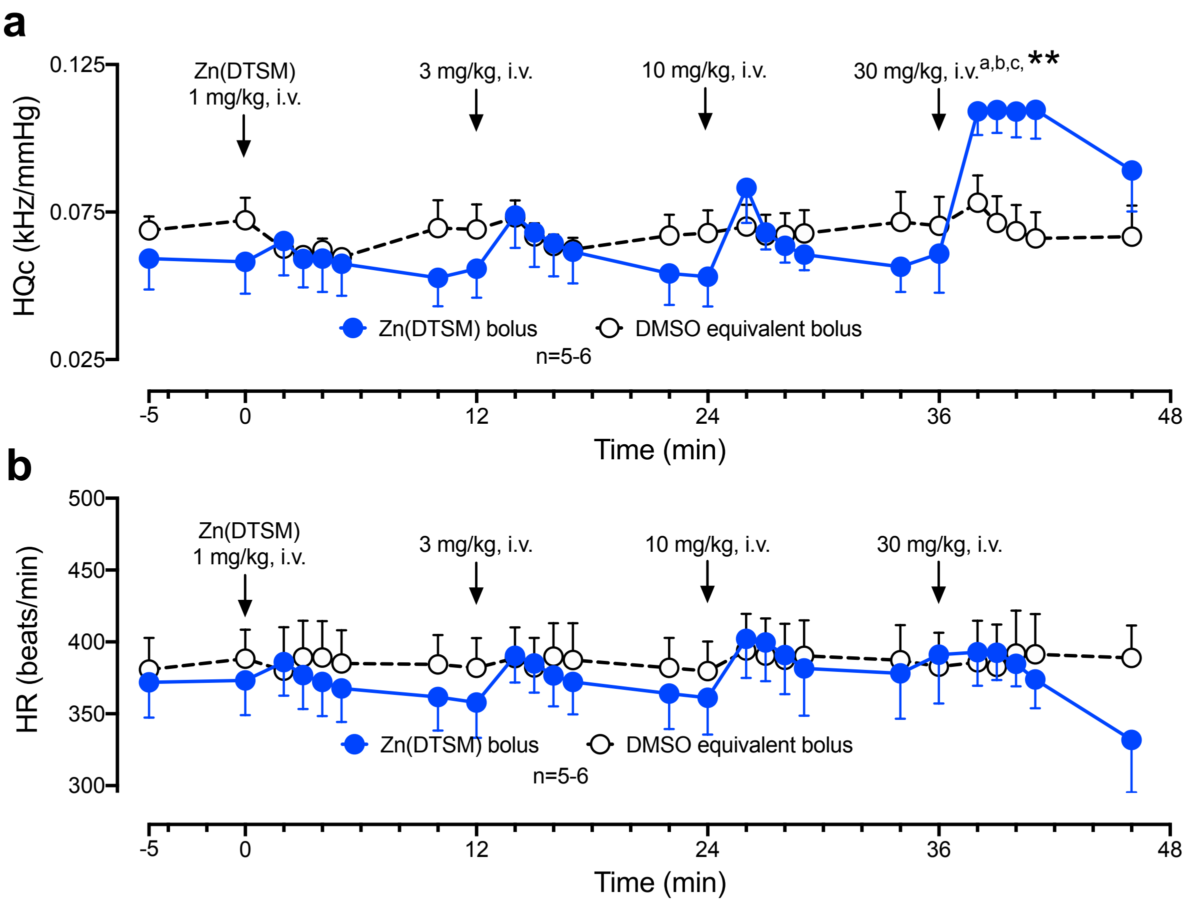

Supplementary Fig. 3: Haemodynamic parameters after Zn(DTSM) bolus intravenous injections in anaesthetized rats. (**a**) Hindquarter vascular conductance (HQc), and (**b**) heart rate (HR) after intravenous (i.v.) bolus injections of 1, 3, 10 and 30 mg/kg Zn(DTSM) or vehicle (DMSO) equivalent (n=5-6 each). The area under the curve within the 10 min post-each dose was used for comparison. ^{a}p=0.0011 compared to 1 mg/kg, ^{b}p=0.0085 compared to 3 mg/kg, ^{c}p=0.034 compared to 10 mg/kg Zn(DTSM) [F(1, 35) = 12.34, p=0.0012], **p=0.0011 compared to the equivalent dose of vehicle, 2-way ANOVA [F(3, 35) = 4.126, p=0.0132] with Sidak's post-test. Responses are expressed as mean ± SEM. n, number of rats; each rat received all four doses.

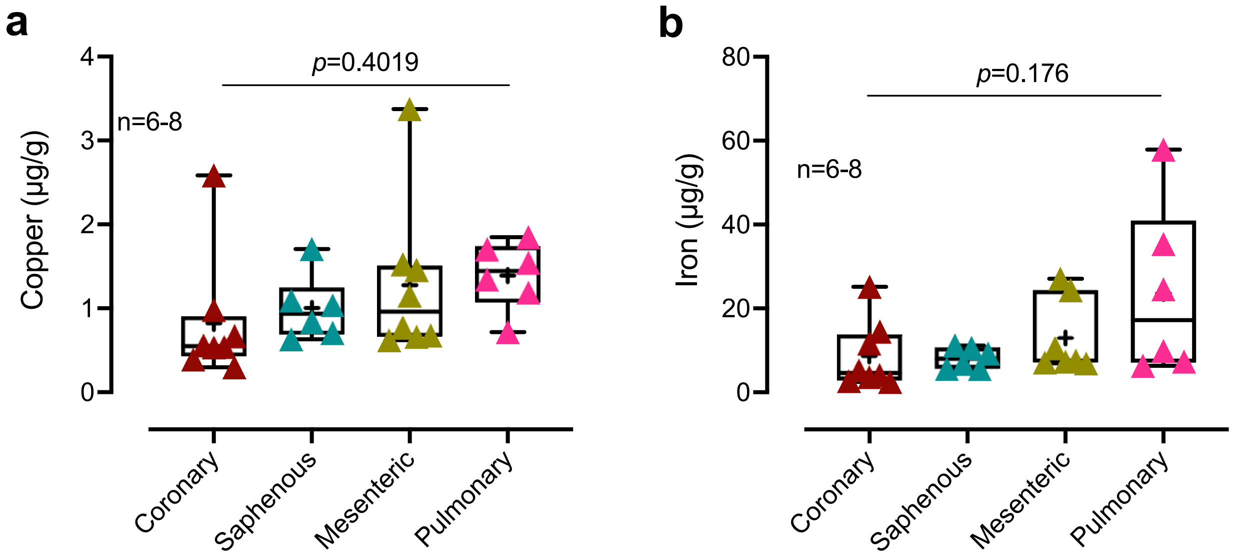

Supplementary Fig. 4: Total copper and iron levels of arteries measured by ICP-MS do not differ. (**a**) Copper and (**b**) iron levels of rat isolated mesenteric, femoral, coronary and pulmonary arteries normalized to the wet weight of the tissues. 1-way ANOVA [$F(3,24)=1.018$, $p=0.4019$] (**a**) or Kruskal-Wallis test [$H(3)=4.396$, $p=0.1765$] (**b**). Whiskers are min to max values and boxes are 25th to 75th percentile, where the line inside the box is the median and the + sign is the mean. n, number of arteries isolated from separate rats.

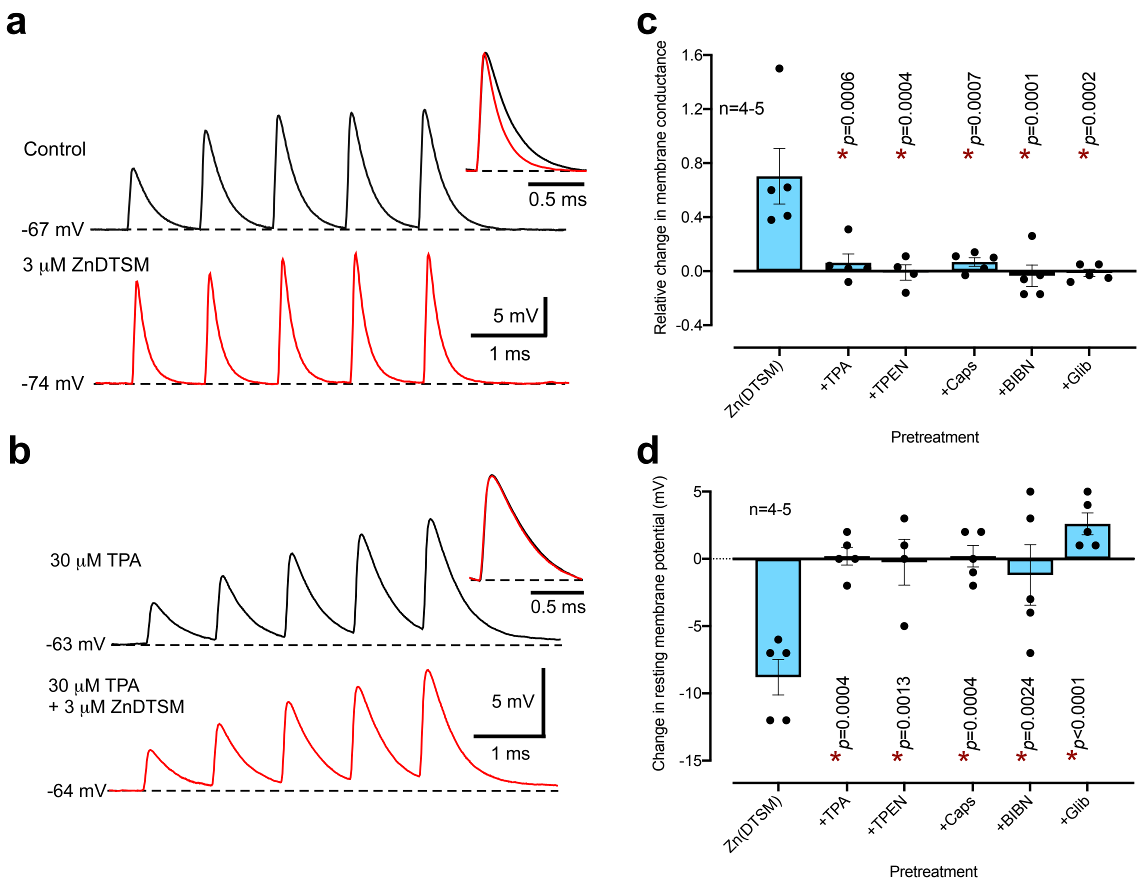

Supplementary Fig. 5: Zn(DTSM) causes hyperpolarization and increases vascular smooth muscle membrane conductance by releasing CGRP from sensory nerves. (**a-b**) Membrane potential and responses to five stimuli at 1 Hz before (control) and during 3 μmol/L, μM, Zn(DTSM). In (**b**), the tissue was pretreated with the intracellular zinc chelator, TPA (30 μM). Insets show amplitude normalized expanded overlaid traces of the 5th EJP before and during Zn(DTSM). (**c-d**) Effects of Zn(DTSM) on membrane conductance (**c**) and resting membrane potential (**d**) were blocked by the intracellular zinc chelators TPA (30 μM) and TPEN (30 μM), pretreatment with capsaicin (1 μM) to desensitize sensory nerves, the CGRP antagonist BIBN4096 (1 μM) or blockade of ATP-sensitive potassium channel by glibenclamide (3 μM). Error bars are SEM. 1-way ANOVA [$F(5, 23)=8.614$, $p=0.0001$ in **c** and $F(5, 23)=8.141$, $p=0.0002$ in **d**] followed by Dunnett's post-test vs Zn(DTSM). Data were collected from n=5 mesenteric arteries from separate rats.

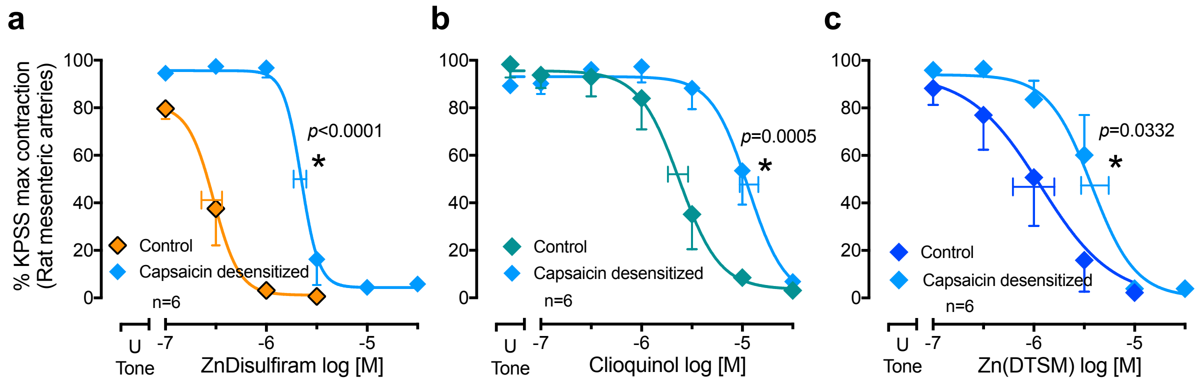

Supplementary Fig. 6: Sensory nerve desensitization decreases the potency of zinc ionophores in rat mesenteric arteries. (**a**-**c**) Cumulative concentration-relaxation curves for zinc disulfiram (ZnDisulfiram) (**a**), clioquinol (**b**) and Zn(DTSM) (**c**) in the absence (control) or presence of capsaicin desensitization (10 μM for 30 min followed by washout) in U46619 contracted (U Tone) arteries. Responses are expressed as a % of the KPSS (124 mM K^+) reference contraction. Vertical error bars are ± SEM and horizontal error bars represent the average EC_{50} ± SEM. n, number of arteries isolated from separate rats. *Statistically significant, two-tailed unpaired Student's *t*-test of EC_{50} [T(10)=7.290 in **a**, T(10)=5.001 in **b** and T(10)=2.468 in **c**].

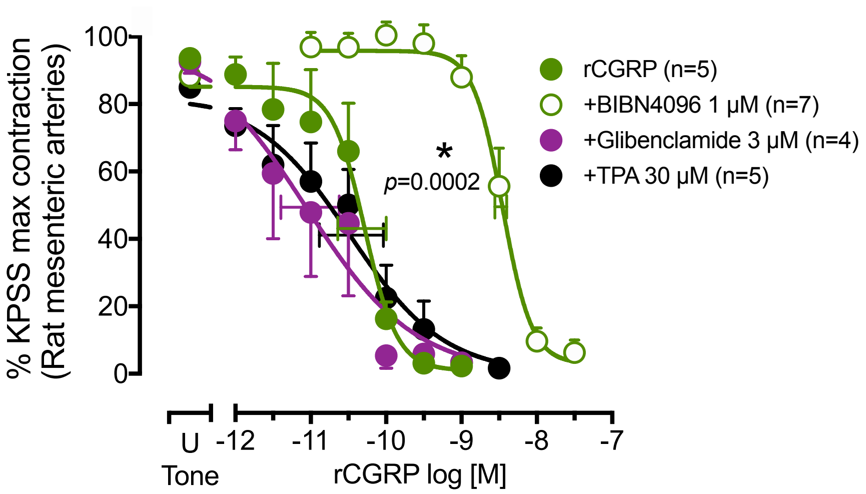

Supplementary Fig. 7: Vasorelaxant effects of rat calcitonin gene-related peptide (rCGRP) in rat mesenteric arteries. Cumulative concentration-relaxation curves for rCGRP in the absence or presence of the CGRP receptor antagonist BIBN4096 (1 μmol/L, μM), K_{ATP} channel inhibitor glibenclamide (3 μM) or the cell-permeable zinc chelator TPA (30 μM). The arteries were contracted with the thromboxane A2-mimetic, U46619. Responses are expressed as a % of the KPSS (124 mM K^+) reference contraction. Vertical error bars are ± SEM and horizontal error bars are the average EC_{50} ± SEM. n, number of arteries isolated from separate rats. ****p*<0.001, 1-way ANOVA [$F(3, 17) = 16.63$, $p<0.0001$] with Dunnett's post-test of the EC_{50} values.

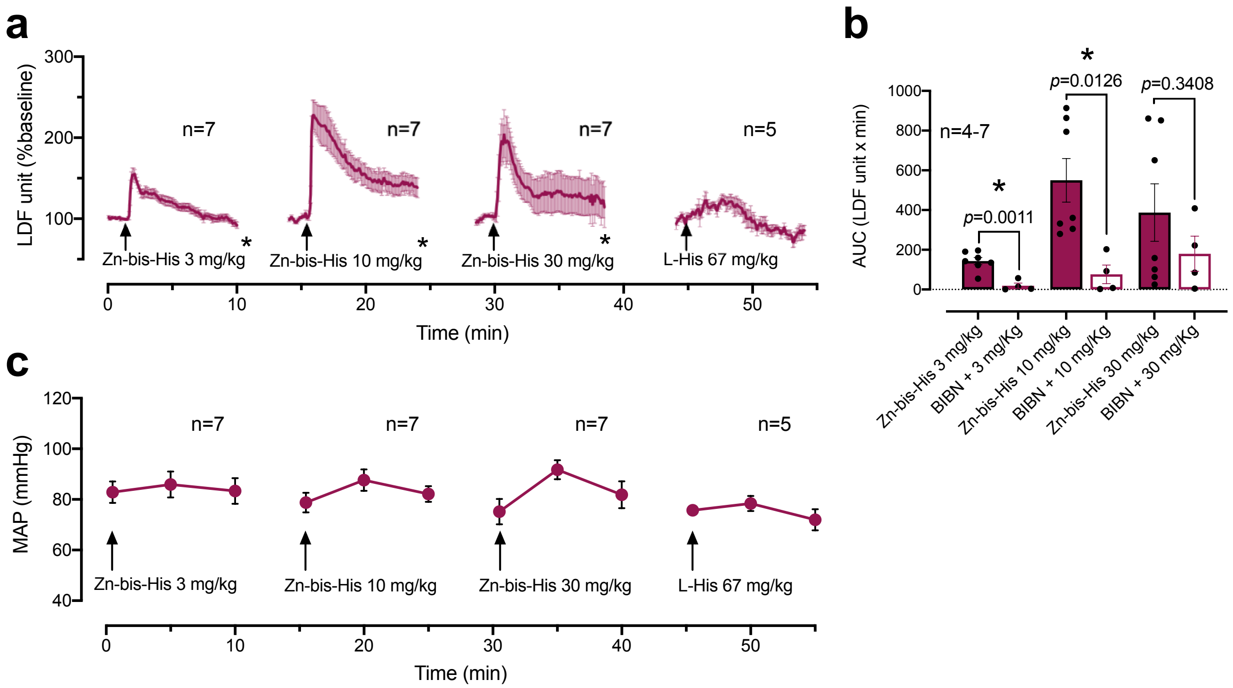

Supplementary Fig. 8: Zn-bis-Histidinate increases cutaneous blood flow after bolus intravenous injection. (**a**) Hind paw cutaneous laser Doppler flux (LDF), (**b**) area under the vasodilatation curve with/without BIBN (3 mg/kg) pretreatment *in vivo* and (**c**) mean arterial pressure (MAP) after an intravenous (i.v.) bolus injection of 3, 10 and 30 mg/kg zinc-bis(histidinate) (Zn-bis-His) or equivalent dose of L-histidine (L-His, 67 mg/kg) for the highest dose of Zn-bis-His tested. Error bars are ± SEM. n, number of rats; All 7 rats studied were used to test all the 3 doses of Zn-bis-his at the time indicated, but only 5 received the subsequent dose of L-His. *Statistically significant, mixed-effects 2-way ANOVA [$F(3,22)=4.010$, $p=0.0203$ for concentration; $F(3.407,72.89)= 23.14$, $p<0.0001$ for time] with Dunnett's post-test (**a**); two-tailed unpaired Student's *t*-test of AUC [($T(9)=4.718$ for 3 mg/kg, $T(9)=3.106$ for 10 mg/kg] (**b**).

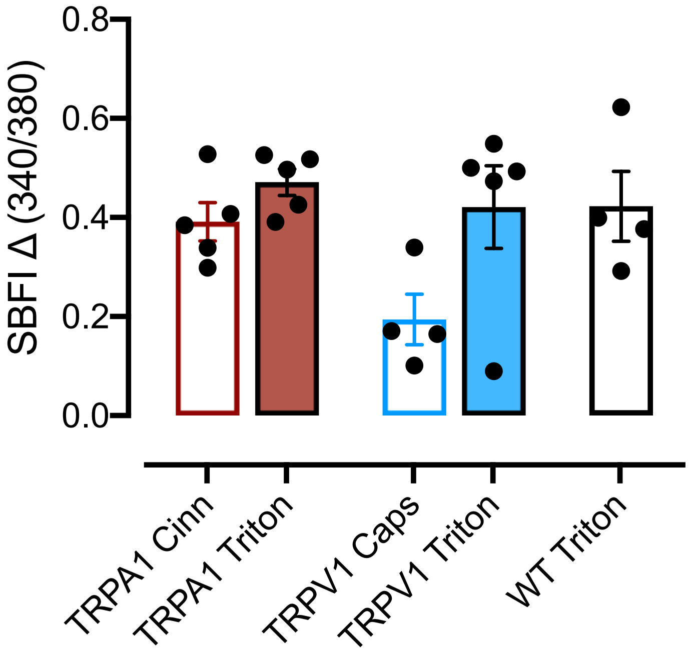

Supplementary Fig. 9: hTRPA1 or hTRPV1 channels expressed in human embryonic kidney (HEK293) cells were activated by known agonists: positive controls for the experiments shown in Fig. 4d. Sodium influx was measured using increase in Na^+-sensitive dye SBFI-AM fluorescence (excitation at 340/380 nm and emission at 505 nm). In TRPA1 or TRPV1 expressing HEK293 cells sodium influx was evoked by cinnamaldehyde (Cinn, 300 µM) and capsaicin (Caps, 3 µM), respectively. In both non-transfected (WT) and transfected HEK293 cells, sodium influx was evoked by membrane permeabilization with 1 % Triton X (Triton). Error bars are ± SEM. n, number of independent experiments from separate days.

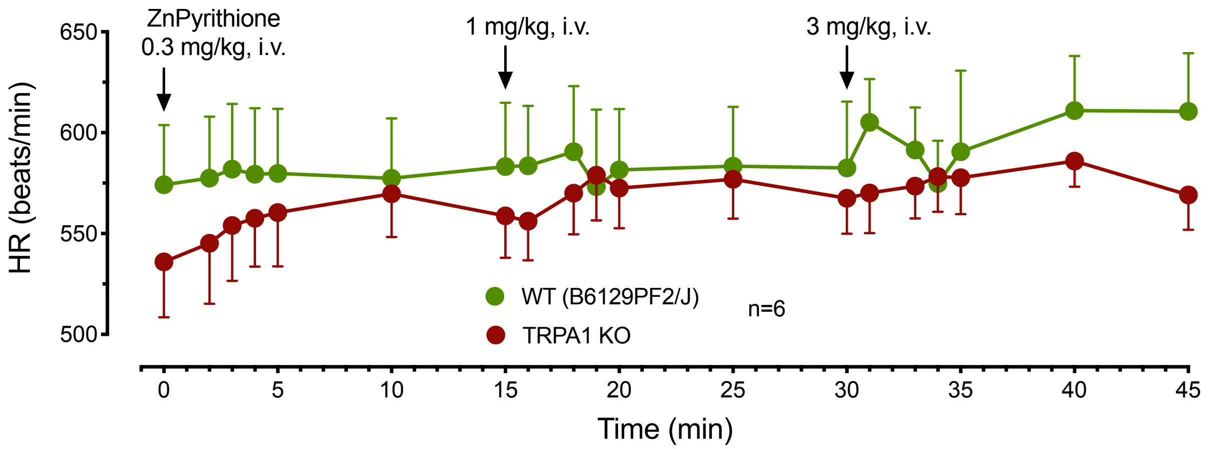

Supplementary Fig. 10: Heart rate measurement after zinc pyrithione bolus intravenous injections in anaesthetized mice. Heart rate (HR) after intravenous (i.v.) bolus injections of 0.3, 1 and 3mg/kg ZnPyrithione in TRPA1 KO or WT (B6129PF2/J) mice. Responses are expressed as mean ± SEM. n, number of mice; each mouse received all three doses.

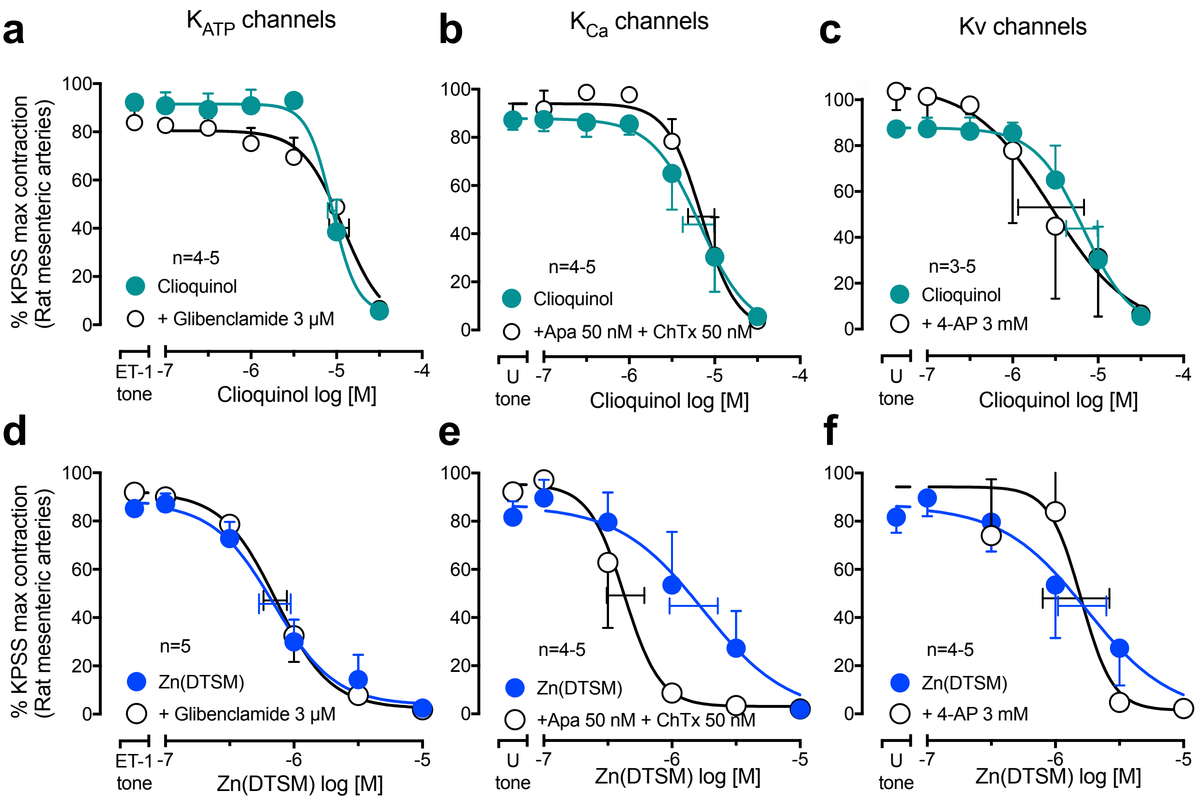

Supplementary Fig. 11: Lack of effect of potassium channel blockers in the vasorelaxant actions of zinc ionophores in rat mesenteric arteries. (**a-f**) Concentration-relaxation curves to clioquinol (**a-c**) or Zn(DTSM) (**d-f**) before and during application of the K_{ATP} channel inhibitor, glibenclamide (**a, d;** 3 μM), the calcium-activated potassium channel (K_{Ca}) inhibitors, apamin (Apa, 50 nM) and charybdotoxin (ChTx; 50 nM) (**b, e**), and the voltage-dependent potassium channel (K_V) blocker, 4-aminopyridine (4-AP, 3 mM) (**c, f**). For (**a** and **d**) the arteries were contracted with endothelin-1 (ET-1 tone), while for the others they were contracted with U46619 (U tone). Responses are expressed as a % KPSS (124 mM K^+) reference contraction. Vertical error bars are ± SEM and horizontal error bars are the average EC_{50} ± SEM. n, number of arteries isolated from separate rats. The relaxation curve without pre-treatment in **c** is the same as **b** for clioquinol and in **e** is the same as **f** for Zn(DTSM).

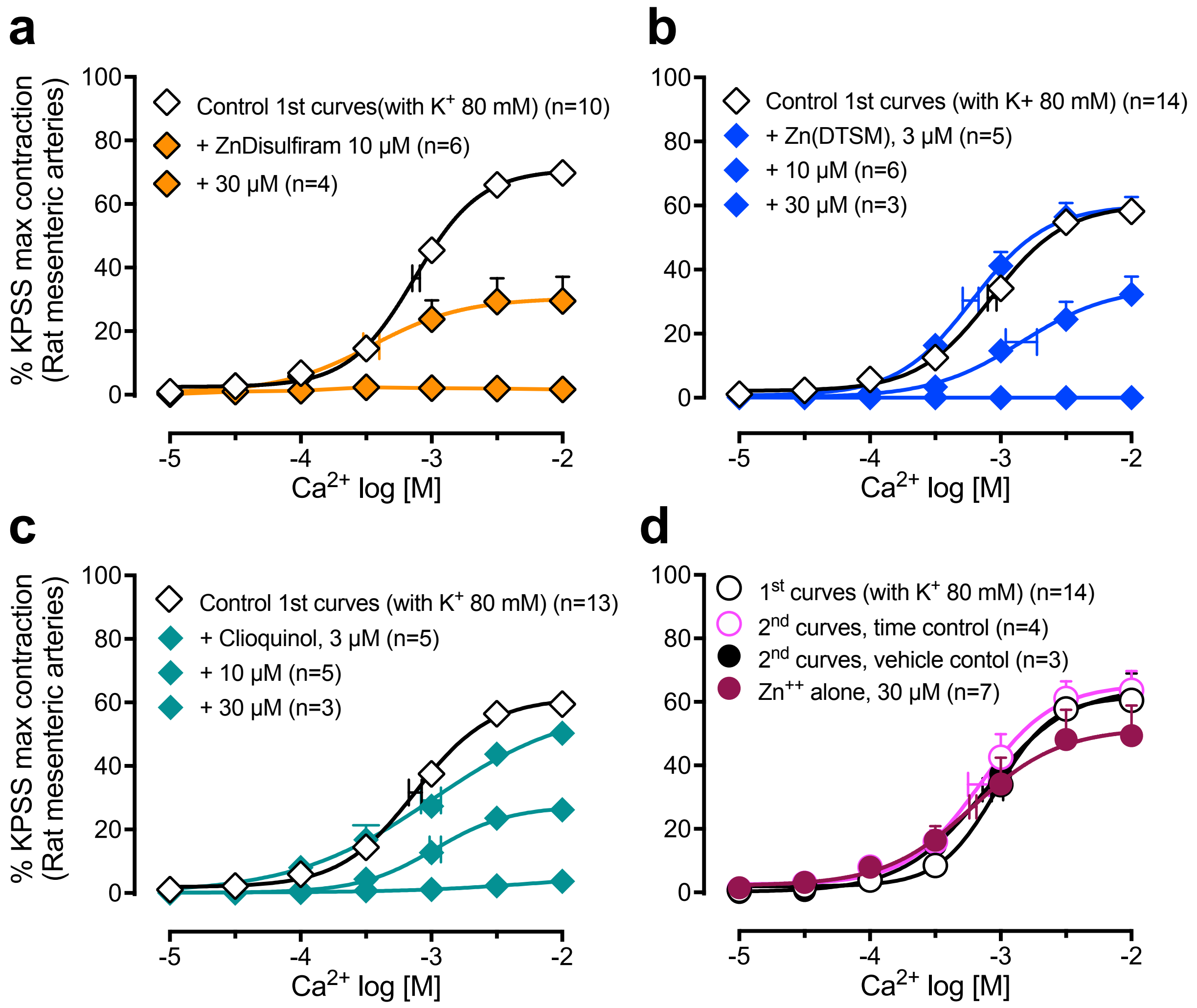

Supplementary Fig. 12: Effects of zinc ionophores on voltage-gated calcium channel-dependent contractions of rat mesenteric arteries. (**a-d**) Concentration-contraction curves for Ca^{2+} in arteries treated with (**a**) zinc disulfiram (ZnDis), (**b**) Zn(DTSM), (**c**) clioquinol or (**d**) Zn^{++} alone. The graphs also show data for vehicle or time controls. In these experiments, depolarization was induced by a high contraction of 80 mM K^+ (in Ca^{2+} free PSS-A) before adding Ca^{2+} to the bathing solution. Responses are expressed as a %KPSS (124 mM K^+) reference contraction. Vertical error bars are ± SEM and horizontal error bars are the average EC_{50} ± SEM. n, number of arteries isolated from separate rats.

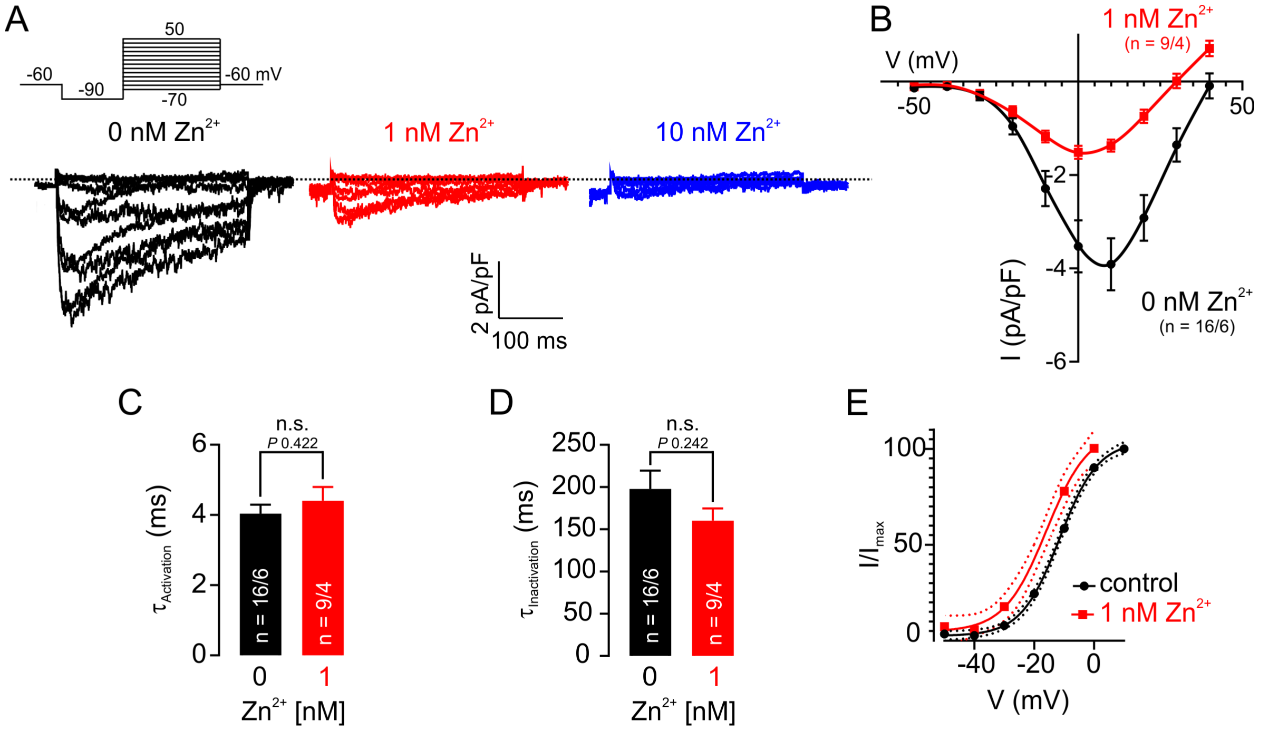

Supplementary Fig. 13: Effects of intracellular zinc on voltage-gated calcium channel (VGCC) activity. (**a**) Representative traces of VGCC currents (Ba^{2+} as the charge carrier) recorded in mesenteric artery smooth muscle cells using conventional whole-cell configuration without (0 nM) or with intracellular zinc (1 nM or 10 nM) directly introduced via the patch pipette. After hyperpolarizing the cell to -90 mV, channel activity was induced using a voltage-step protocol (-70 to +50 mV, upper inset). Traces in (**A**) show currents evoked by steps from -50 to 30 mV. (**b**) Averaged current-voltage (I-V) plots for the VGCC currents in the absence (0 nM Zn^{2+}) or presence of 1 nM Zn^{2+}. (**c**) Time constants (τ) of activation and (**d**) inactivation without or with intracellular Zn^{2+} (1 nM). (**e**) Averaged voltage dependence of activation in the absence or in the presence of 1 nM intracellular Zn^{2+}. Average normalized data (%I/I_{max}) ± SEM were fit using a single Boltzmann distribution (see Methods). Error bars are ± SEM. n.s., not significant, two-tailed unpaired Student's *t*-test. n/n indicates number of cells/number of mice.

Supplementary Table 1. The KPSS reference contraction, *p*EC$_{50}$ and *I*$_{max}$ for concentration-relaxation curves to zinc ionophores (and extracellular Zn^{++}) in arteries isolated from rats and humans.

Tissue/Treatment	Maximum KPSS reference contraction (mN)	*p*EC$_{50}$	Baseline tone (%KPSS)	*I*$_{max}$ (%KPSS)	n
**Rat mesenteric arteries**					
Acetylcholine	8.7 ± 0.6	6.99 ± 0.17	93 ± 3	2 ± 1	5
Zinc Disulfiram	10 ± 0.4	6.54 ± 0.10	81 ± 2	1 ± 0.4	6
Zinc Pyrithione	8.5 ± 0.8	6.44 ± 0.08	93 ± 3	2 ± 0.5	7
Zn(DTSM)	9.8 ± 0.8	6.08 ± 0.21	95 ± 4	2 ± 0.4	6
Clioquinol	11 ± 1.1	5.36 ± 0.17	89 ± 4	5 ± 1	6
Zn-bis-Histidinate	10 ± 0.4	4.18 ± 0.09	94 ± 3	3 ± 0.3	6
Zn^{++}	10 ± 0.8	N/A	84 ± 4	61 ± 12	6
**Zinc Pyrithione in rat arteries**					
Mesenteric	12 ± 0.3	6.55 ± 0.09	95 ± 4	2 ± 1	8
Middle cerebral	9.4 ± 0.8	5.29 ± 0.09	104 ± 2	3 ± 0.4	6
Basilar	13 ± 1.0	5.52 ± 0.16	105 ± 5	5 ± 1	6
Coronary	4.3 ± 0.4	4.73 ± 0.03	90 ± 5	3 ± 0.4	6
Saphenous	16 ± 1.3	5.26 ± 0.15	80 ± 3	4 ± 1	6
Pulmonary	17 ± 3.9	4.61 ± 0.10	79 ± 3	13 ± 2	6
Renal	9.8 ± 0.4	5.01 ± 0.07	83 ± 3	4 ± 1	6
Aorta	16 ± 2.1	4.64 ± 0.07	92 ± 2	15 ± 2	6
**Zinc ionophores in rat saphenous vein**					
Zinc Pyrithione	6.2 ± 0.8	5.33 ± 0.16	86 ± 7	5 ± 1	4
Zn(DTSM)	6.1 ± 0.5	5.49 ± 0.05	83 ± 6	6 ± 1	6
Clioquinol	5.6 ± 0.9	4.86 ± 0.12	84 ± 3	5 ± 1	5
**Human internal mammary artery**					
Zinc Pyrithione	29 ± 4.6	5.07 ± 0.13	73 ± 3	12 ± 4	8
Zn(DTSM)	33 ± 5.9	5.11 ± 0.05	78 ± 3	9 ± 3	7
Zn^{++} alone	51 ± 11	N/A	72 ± 2	53 ± 8	6
**Human saphenous vein**					
Zn(DTSM)	36 ± 10	4.96 ± 0.12	80 ± 4	14 ± 4	6
Zn^{++} alone	42 ± 15	N/A	80 ± 5	64 ± 8	5

Responses were tested after arteries were contracted with U46619 (Baseline tone). I_{max}, the maximum inhibition responses produced expressed as a % of the maximum KPSS reference contraction; pEC_{50}, $-\log_{10}EC_{50}$; Values are mean ± 1 SEM. N/A, not applicable; n, number of arteries isolated from separate rats or humans.

Supplementary Table 2. Effects of pyrithione or various agents on the resting membrane potential (RMP) and decay time constants of excitatory junction potentials (τEJP).

Compound used (concentration)	RMP (mV)			τEJP (ms)			n
	Control	Treatment	*p* value	Control	Treatment	*p* value	
Pyr (3 μM)	-67 ± 1	-75 ± 1*	0.003	296 ± 22	147 ± 5*	<0.001	6
TPA (30 μM)	-65 ± 1	-59 ± 1*	0.014	271 ± 16	315 ± 19*	0.033	6
Glibenclamide (3μM)	-67 ± 1	-59 ± 1*	0.001	237 ± 11	375 ± 18*	<0.001	6
Capsaicin (10 μM)	-66 ± 2	-65 ± 1†	0.71	303 ± 41	337 ± 43	0.09	6
BIBN4096 (1 μM)	-67 ± 2	-61 ± 1*	0.003	238 ± 13	368 ± 19*	<0.001	6
HC030031 (50 μM)	-65 ± 1	-65 ± 2	0.83	282 ± 12	314 ± 20*	0.033	6
Ruthenium Red (10 μM)	-68 ± 1	-67 ± 1	0.25	279 ± 9	373 ± 20*	0.005	6
Capsazepine (10 μM)	-66 ± 1	-67 ± 1	0.093	294 ± 17	284 ± 18	0.35	6
Zn^{++} (100 μM)	-67 ± 1	-68 ± 1	0.36	291 ± 19	265 ± 11	0.14	5

†Value after 20 min washout of capsaicin. RMP and EJPs were recorded in the smooth muscle cells of rat isolated mesenteric arteries without (Control) or with pyrithione (Pyr 3 μM), Zn^{++} (100 μM) or various inhibitors of different pathways used as pre-treatments in Fig 2 and Fig S7. EJPs were evoked by trains of five stimuli at 1 Hz and the τEJPs were computed from the 5th EJP in the train. Values are mean ±1 SEM. N/A, not applicable; n, number of arteries isolated from separate rats. *$p<0.05$, two-tailed paired Student's *t*-test compared to control.

Supplementary Table 3. The KPSS reference contraction, pEC_{50} and I_{max} for concentration-relaxation curves to zinc ionophores without or with inhibitors of vasoactive mediators from sensory nerves or endothelium.

Treatment	Maximum KPSS contraction (mN)	pEC_{50}	p value	Baseline tone (%KPSS)	I_{max} (%KPSS)	n
**Zinc Pyrithione**						
Control	11.7 ± 0.3	6.55 ± 0.09		95 ± 4	1 ± 0.3	8
+ Capsaicin desensitized (10 μM)	8.3 ± 0.8	5.14 ± 0.05	<0.0001	89 ± 4	4 ± 0.6	6
+ Capsaicin desensitization + TPA (100 μM)	9.1 ± 0.8	N/A		97 ± 5	72 ± 5	6
+ BIBN4096 (1 μM)	10.4 ± 0.8	5.35 ± 0.10	<0.0001	87 ± 7	4 ± 0.5	6
- Endothelium (by denudation)	10.2 ± 0.6	5.77 ± 0.14	<0.0001	93 ± 3	3 ± 0.8	8
- Endothelium (by denudation) + Capsaicin desensitization	11.7 ± 0.7	4.97 ± 0.07	<0.0001	96 ± 6	5 ± 1	6
+ Indomethacin (3 μM)	12.8 ± 0.4	6.09 ± 0.14	0.022	96 ± 3	1 ± 0.5	9
+ L-NAME (100 μM)	12 ± 1	6.54 ± 0.11	>0.999	103 ± 4	1 ± 0.6	8
+ L-NAME (100 μM) + Indomethacin (3 μM)	10.6 ± 0.8	5.67 ± 0.14	<0.0001	96 ± 5	3 ± 1	7
+ ODQ (3 μM)	10.7 ± 0.3	6.69 ± 0.06	0.97	102 ± 6	1 ± 0.4	6
+ Apamin (50 nM) + Charybdotoxin (50 nM)	11.7 ± 0.9	6.45 ± 0.13	0.99	90 ± 4	1 ± 0.3	7
**Zinc Pyrithione**						
Control	11.1 ± 0.3	6.40 ± 0.10		88 ± 4	2 ± 0.3	6
+ HC030031 (50 μM)	10.1 ± 0.2	5.94 ± 0.12	0.0061	75 ± 2	2 ± 0.7	6
+ AM0902 (10 μM)	10.2 ± 0.5	5.70 ± 0.06	0.0001	84 ± 4	1 ± 0.7	6
+ Ruthenium Red (10 μM)	10.7 ± 0.6	5.57 ± 0.08	<0.0001	91 ± 3	2 ± 0.4	6
**Zinc disulfiram**						
Control	10 ± 0.4	6.54 ± 0.10		81 ± 2	2 ± 1	6
+ capsaicin desensitization	7.6 ± 0.3	5.67 ± 0.06	<0.0001	93 ± 4	4 ± 1	6
- endothelium (by denudation)	11.1 ± 1	6.24 ± 0.13	0.102	103 ± 8	1 ± 1	6
**Clioquinol**						
Control	8.2 ± 0.5	5.64 ± 0.10		98 ± 5	3 ± 1	6
+ Capsaicin desensitization	7.8 ± 0.3	5.00 ± 0.08	0.0003	89 ± 3	7 ± 1	6
- Endothelium (by denudation)	11.2 ± 0.6	5.26 ± 0.09	0.021	105 ± 10	2 ± 1	5
**ZnDTSM**						
Control	9.8 ± 0.8	6.08 ± 0.21		95 ± 4	2 ± 0.4	6
+ Capsaicin desensitization	10 ± 1	5.47 ± 0.14	0.028	94 ± 2	4 ± 1	6
- Endothelium (by denudation)	9.9 ± 0.8	5.68 ± 0.12	0.151	85 ± 2	2 ± 0.4	6

Responses were tested after arteries were contracted with U46619 (Baseline tone). I_{max}, the maximum inhibition responses produced expressed as a % of the maximum KPSS reference contraction; pEC$_{50}$, -log$_{10}$EC$_{50}$; Values are mean ± 1 SEM. N/A, not applicable; n, number of arteries isolated from separate rats. p values are from Dunnett's post-test compared to control after 1-way ANOVA (zinc pyrithione) or two-tailed paired Student's t-test compared to control (zinc disulfiram, clioquinol and ZnDTSM).

Supplementary References

1. Angus JA, Wright CE. Techniques to study the pharmacodynamics of isolated large and small blood vessels. *J. Pharmacol. Toxicol. Methods*. 2000;44:395-407
2. Ayton S, Portbury S, Kalinowski P, Agarwal P, Diouf I, Schneider JA, Morris MC, Bush AI. Regional brain iron associated with deterioration in alzheimer's disease: A large cohort study and theoretical significance. *Alzheimers Dement*. 2021
