# Supplementary material for: Zinc drives vasorelaxation by acting in sensory nerves, endothelium and smooth muscle
Source: Nat Commun. 2021 Jun 1;12:3296. doi: 10.1038/s41467-021-23198-6 (PMC8169932; doi:10.1038/s41467-021-23198-6)
Supplement: Supplementary file 2 — Reporting Summary [file 41467_2021_23198_MOESM2_ESM.pdf]

Corresponding author(s): Scott Ayton, Christine Wright

Last updated by author(s): Apr 9, 2021

Reporting Summary

Nature Research wishes to improve the reproducibility of the work that we publish. This form provides structure for consistency and transparency in reporting. For further information on Nature Research policies, see our Editorial Policies and the Editorial Policy Checklist.

Statistics

For all statistical analyses, confirm that the following items are present in the figure legend, table legend, main text, or Methods section.

n/a	Confirmed	
☐	☒	The exact sample size (n) for each experimental group/condition, given as a discrete number and unit of measurement
☐	☒	A statement on whether measurements were taken from distinct samples or whether the same sample was measured repeatedly
☐	☒	The statistical test(s) used AND whether they are one- or two-sided<br>*Only common tests should be described solely by name; describe more complex techniques in the Methods section.*
☒	☐	A description of all covariates tested
☐	☒	A description of any assumptions or corrections, such as tests of normality and adjustment for multiple comparisons
☐	☒	A full description of the statistical parameters including central tendency (e.g. means) or other basic estimates (e.g. regression coefficient) AND variation (e.g. standard deviation) or associated estimates of uncertainty (e.g. confidence intervals)
☐	☒	For null hypothesis testing, the test statistic (e.g. F, t, r) with confidence intervals, effect sizes, degrees of freedom and P value noted<br>*Give P values as exact values whenever suitable.*
☒	☐	For Bayesian analysis, information on the choice of priors and Markov chain Monte Carlo settings
☒	☐	For hierarchical and complex designs, identification of the appropriate level for tests and full reporting of outcomes
☒	☐	Estimates of effect sizes (e.g. Cohen's d, Pearson's r), indicating how they were calculated

Our web collection on statistics for biologists contains articles on many of the points above.

Software and code

Policy information about availability of computer code

Data collection	LabChart 7, LabChart 8, Igor Pro 6.3, MyoDaq 2.01, Myodata2.01
Data analysis	GraphPad Prism 8, Igor Pro 6.3, Clampfit 10.3, Maxchelator

For manuscripts utilizing custom algorithms or software that are central to the research but not yet described in published literature, software must be made available to editors and reviewers. We strongly encourage code deposition in a community repository (e.g. GitHub). See the Nature Research guidelines for submitting code & software for further information.

Data

Policy information about availability of data

All manuscripts must include a data availability statement. This statement should provide the following information, where applicable:
- Accession codes, unique identifiers, or web links for publicly available datasets
- A list of figures that have associated raw data
- A description of any restrictions on data availability

The data supporting the findings of this study are included in the paper and the supplementary file. All other data used in this study are available on request. Source data are provided with this paper.

Field-specific reporting

Life sciences study design

All studies must disclose on these points even when the disclosure is negative.

Sample size	Sample sizes of 5 or more per treatment group were used to allow sound statistical analysis based on standard good in vitro and in vivo pharmacological study design (Curtis et al. Br J Pharmacol 175, 987-993, 2018) and our extensive experience with similar work (Betrie et al. J Alzheimers Dis 56, 849-860, 2017; He et al. Ann Thorac Surg 55, 1210-1217, 1993; Angus et al. Eur J Pharmacol 750, 43–51, 2015; Dunn et al. Br J Pharmacol 140, 231-238, 2003)
Data exclusions	No data presented in the study was excluded from analysis.
Replication	Recordings or measurements were successfully replicated using 5 or more independent samples per treatment group to ascertain reproducibility of the findings.
Randomization	Treatments were randomly assigned to each isolated artery preparation or animal on the day of experiment using simple randomization technique.
Blinding	Investigators were not blinded to group allocation for this study as experimental design and data collection was performed by the same individuals that allocate the groups. However all the data collected was functional physiological/numerical data with lower probability for bias.

Reporting for specific materials, systems and methods

We require information from authors about some types of materials, experimental systems and methods used in many studies. Here, indicate whether each material, system or method listed is relevant to your study. If you are not sure if a list item applies to your research, read the appropriate section before selecting a response.

Materials & experimental systems

n/a	Involved in the study	
☒	☐	Antibodies
☐	☒	Eukaryotic cell lines
☒	☐	Palaeontology and archaeology
☐	☒	Animals and other organisms
☐	☒	Human research participants
☒	☐	Clinical data
☒	☐	Dual use research of concern

Methods

n/a	Involved in the study	
☒	☐	ChIP-seq
☒	☐	Flow cytometry
☒	☐	MRI-based neuroimaging

Eukaryotic cell lines

Policy information about cell lines

Cell line source(s)	T-Rex-293 Human embryonic kidney 293 (HEK293) FlpIn cells, Invitrogen, ThermoFisher Scientific
Authentication	We did not authenticate the cell lines.
Mycoplasma contamination	All cell lines tested negative for Mycoplasma contamination
Commonly misidentified lines (See ICLAC register)	No commonly misidentified cell lines were used in this study.

Animals and other organisms

Policy information about studies involving animals; ARRIVE guidelines recommended for reporting animal research

Laboratory animals	Sprague Dawley rats, male, 250–350 g, 8–9 weeks old; C57BL/6J mice, male, 25-30 g, 8-12 weeks old; TRPA1 KO (B6;129P-Trpa1tm1Kykw/J) mice, male, 25-30 g, 8-12 weeks old; B6129PF2/J mice, male, 25-30 g, 8-12 weeks old
Wild animals	No wild animals were used in this study.
Field-collected samples	No field-collected samples were used in this study.
Ethics oversight	Animal ethic for the protocol was approved by the University of Melbourne Animal Ethics Committee (1212630, 1513798.1 and 1413363.1) or the University of Vermont Institutional Animal Care and Use committee (protocol 18-004).

Note that full information on the approval of the study protocol must also be provided in the manuscript.

Human research participants

Policy information about studies involving human research participants

Population characteristics	Internal mammary artery and saphenous vein was collected from excess discarded tissues of 14 patients (8 males, 6 females), 55 – 75 years old, undergoing routine coronary artery bypass graft surgery.
Recruitment	All patients undergoing routine coronary artery bypass graft surgery at the TEDA International Cardiovascular Hospital, Tianjin, China during the study period that gave consent (14) were recruited hence avoiding selection bias.
Ethics oversight	All human protocols and use of tissues were approved by the Institutional Review Board of TEDA International Cardiovascular Hospital (TICH, ethics approval no: [2016]-1219-2 and [2014]-1223-4)

Note that full information on the approval of the study protocol must also be provided in the manuscript.
